# Isotopic labelling reveals the efficient adaptation of wheat root TCA cycle flux modes to match carbon demand under ammonium nutrition

**DOI:** 10.1038/s41598-019-45393-8

**Published:** 2019-06-20

**Authors:** Izargi Vega-Mas, Caroline Cukier, Inmaculada Coleto, Carmen González-Murua, Anis M. Limami, M Begoña González-Moro, Daniel Marino

**Affiliations:** 1 Department of Plant Biology and Ecology, University of the Basque Country (UPV/EHU), Apdo. 644, E-48080 Bilbao, Spain; 2 University of Angers, Institut de Recherche en Horticulture et Semences, INRA, Structure Fédérative de Recherche 4207, Qualité et Santé du Végétal, F-49045 Angers, France; 3 Ikerbasque, Basque Foundation for Science, E-48011 Bilbao, Spain

**Keywords:** Metabolomics, Plant physiology, Mass spectrometry

## Abstract

Proper carbon (C) supply is essential for nitrogen (N) assimilation especially when plants are grown under ammonium (NH_4_^+^) nutrition. However, how C and N metabolic fluxes adapt to achieve so remains uncertain. In this work, roots of wheat (*Triticum aestivum* L.) plants grown under exclusive NH_4_^+^ or nitrate (NO_3_^−^) supply were incubated with isotope-labelled substrates (^15^NH_4_^+^, ^15^NO_3_^−^, or [^13^C]Pyruvate) to follow the incorporation of ^15^N or ^13^C into amino acids and organic acids. Roots of plants adapted to ammonium nutrition presented higher capacity to incorporate both ^15^NH_4_^+^ and ^15^NO_3_^−^ into amino acids, thanks to the previous induction of the NH_4_^+^ assimilative machinery. The ^15^N label was firstly incorporated into [^15^N]Gln vía glutamine synthetase; ultimately leading to [^15^N]Asn accumulation as an optimal NH_4_^+^ storage. The provision of [^13^C]Pyruvate led to [^13^C]Citrate and [^13^C]Malate accumulation and to rapid [^13^C]2-OG consumption for amino acid synthesis and highlighted the importance of the anaplerotic routes associated to tricarboxylic acid (TCA) cycle. Taken together, our results indicate that root adaptation to ammonium nutrition allowed efficient assimilation of N thanks to the promotion of TCA cycle open flux modes in order to sustain C skeleton availability for effective NH_4_^+^ detoxification into amino acids.

## Introduction

Metabolic networks are generally considered as more complex in plants compared to other organisms. This complexity is due to several aspects, which include the huge chemical repertoire of plants together with their sessile nature that makes them to be adaptable to a range of environmental conditions, including the nutritional heterogeneity of soils. Nitrogen (N) is an essential nutrient for plant growth and development, and plants need to rearrange their metabolism in function of both the availability and the form of N present in the soil^1,2^. The typical N deficiency of agricultural soils is behind the need of the supply of nitrogenous fertilizers to maintain optimal crop yield. In this context, sustainable agriculture pursues to maximise nitrogen use efficiency (NUE) to reduce the cost of fertilization and its environmental impacts. NUE depends, among others, on N uptake and assimilation efficiency, which both rely on the N form available in the soil^3,4^.

In general, the most common available N forms are nitrate (NO_3_^−^) and ammonium (NH_4_^+^). Their proportions are dependent on environmental conditions, on soil microbiota and on soil use and management. Although plants are able to use both N forms, the preference to absorb one or the other varies between species and even within the same species. In any case, when NH_4_^+^ is present in high concentrations it provokes a number of physiological and morphological disturbances collectively known as ammonium syndrome^5^. The symptoms associated to this syndrome include alterations in pH homeostasis, ionic imbalance, alterations in plant development and a profound adaption of cell metabolism. Ultimately, ammonium syndrome may entail a severe stressful situation provoking a dramatic biomass reduction, chlorosis and even plant death^5,6^. One of the main strategies that plants deploy to avoid excessive NH_4_^+^ accumulation in tissues is its assimilation into organic molecules, principally through GS/GOGAT pathway^7^. The induction of GS activity is a common response of plants when facing ammonium nutrition^8,9^ and *Arabidopsis thaliana* mutants lacking GLN1:2 isoform display ammonium accumulation and hypersensitivity to ammonium stress^10^. Indeed, GS enzyme has been considered as a marker to predict the N status in many plant species including wheat^11,12^.

NH_4_^+^ is a highly mobile molecule and it is present in the xylem sap, therefore it can be assimilated in leaves^13,14^. However, in general, roots are the primary site of ammonium assimilation. Indeed, the root is the first organ facing high NH_4_^+^ concentrations in the medium and acts as a physiological barrier to prevent its transport to the more sensitive shoot, where the excess of NH_4_^+^ can impair photosynthetic apparatus^5,6^. In this line, the metabolic adjustment of roots to exclusive ammonium nutrition has even been shown to determine the capacity of the plants to cope with ammonium stress in many species including wheat and tomato^15,16^. Interestingly, in other species such as oilseed rape (*Brassica napus*) the metabolic adjustment seems to occur mostly at the leaf level^17^. Proper carbon (C) supply to maintain ammonium assimilation in the roots is considered as a key aspect to deal with an excess of NH_4_^+^ ^18^. In this sense, the provision of exogenous inorganic C to the root zone partially increased ammonium tolerance in cucumber and tomato^19–21^. Similarly, biomass reduction observed in tomato roots under ammonium stress did not happen when plants were grown at high atmospheric CO_2_ conditions^15^, therefore evidencing the important interplay between N and C metabolism in roots exposed to ammonium nutrition.

The supply of C skeletons is mediated by the tricarboxylic acid (TCA) cycle, together with its associated anaplerotic routes, which becomes essential for N assimilation into amino acids^22^. Actually, Arabidopsis mutants with reduced PEPC or ICDH activity showed alteration in the synthesis of amino acids^23,24^. Moreover, several works have reported an increase of *in vitro* determined TCA-associated enzyme activities when plants deal with ammonium stress, suggesting that the provision of C skeletons linked to TCA is essential to maintain NH_4_^+^ homeostasis^9,25–27^. Indeed, the high C demand in roots for primary ammonium assimilation has been put forward as a trade-off of the consumption of C resources responsible for the growth inhibition typically observed under ammonium stress^5^. Nevertheless, there is still a need to understand the *in vivo* adaptation of C and N metabolic fluxes when plants grow under ammonium nutrition compared to nitrate nutrition.

The use of isotope-labelled substrates to study metabolic fluxes is an excellent strategy to obtain a dynamic *in vivo* picture of cell metabolic activity and has been useful to understand different aspects of plant metabolism, including N assimilation and C allocation, mainly through ^13^C and ^15^N labelling^28^. In fact, the sometimes lack of correlation between the information provided by *in vitro* enzyme activities and metabolite data and the poor understanding of the role and the regulation of some enzymes in different cell metabolic contexts urge the use of labelled substrates to track the metabolic fluxes^29,30^.

To further advance in the *in vivo* metabolic strategies root cells deploy to control NH_4_^+^ levels and to adapt to changing N sources, in this work we aimed at understanding 1) how the adaptation of wheat plants to the exclusive provision of nitrate or ammonium as N source determines the efficiency to assimilate one or another N source and 2) how the *in vivo* N assimilation dynamics are linked to TCA cycle activity in the roots. To do so, roots were incubated with either ^15^NH_4_^+^, ^15^NO_3_^−^, or [^13^C]Pyr from thirty minutes to a maximum of six hours; and the enrichment of ^13^C or ^15^N amino acids and organic acids was evaluated by gas chromatography coupled to mass spectrometry.

## Results

### Wheat physiologic and metabolic response to the growth under ammonium or nitrate nutrition

**Figure 1:**
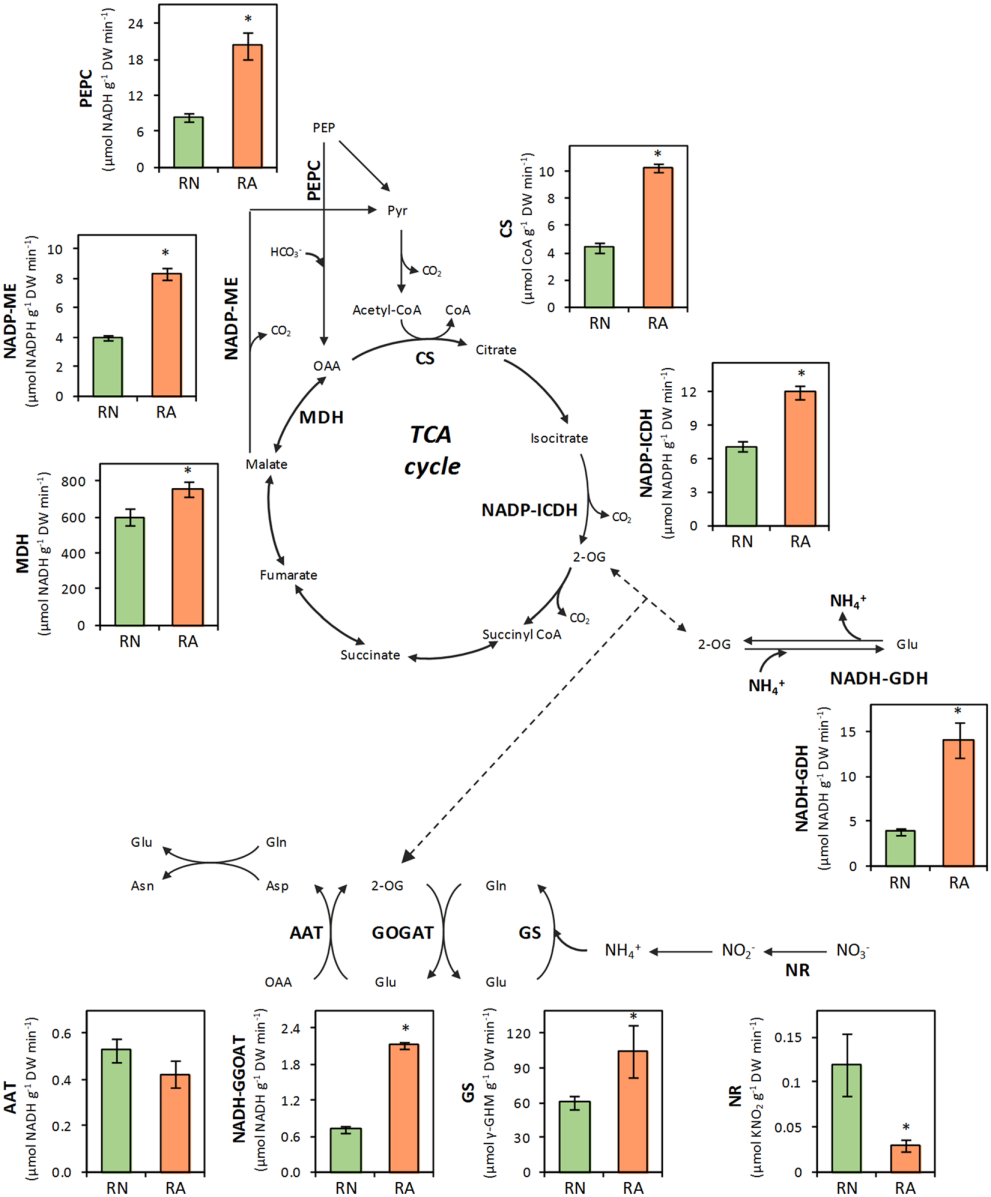
Enzyme activities from primary N assimilation, TCA (tricarboxylic acid) cycle and its associated anaplerotic activities in wheat roots grown under nitrate (RN; green) or ammonium (RA; orange) nutrition. AAT means aspartate aminotransferase, CS citrate synthase, GOGAT glutamate synthase, GS glutamine synthetase, NADH-GDH glutamate dehydrogenase, NADP-ICDH isocitrate dehydrogenase, NADP-ME malic enzyme, NR nitrate reductase, MDH malate dehydrogenase and PEPC phosphoenolyruvate carboxylase. Values represent mean ± SE (n = 6). Asterisk (*) indicates significant differences between N nutritions (p < 0.05).

Most plants, including wheat, have been previously shown to accumulate less biomass when grow with NH_4_^+^ as N source compared as when grow with NO_3_^−^^6,16,31^. Thus, as expected, wheat plants grown under 10 mM ammonium nutrition showed lower biomass production compared with nitrate nutrition (Supplementary Fig. S1), being this effect evident in both shoot and root (Table 1). Nonetheless, the higher chlorophyll contents in the leaf (Table 1) indicated that plants were experiencing a mild stress degree in response to NH_4_^+^ supply. Consistently, ammonium nutrition led to an increased NH_4_^+^, amino acids, protein and C contents in the root compared to nitrate nutrition (Table 1). Asn, Gln and Ala were the major amino acids in wheat roots, their contents being superior in RA with respect to RN (Fig. S2; RN and RA stand for root of plants grown for six weeks under nitrate or ammonium nutrition, respectively). The organic acids contents, in contrast, were higher in RN, and malate and citrate comprised the majority of the organic acids accumulated (Supplementary Fig. S2). As expected, it was observed a general induction of *in vitro* determined root enzyme activities associated with ammonium assimilation (GS, NADH-GOGAT) as well as NADH-GDH activity in RA (Fig. 1). As expected, nitrate reductase (NR) showed an opposite behaviour, being more active under nitrate nutrition. The enzyme activities of the TCA cycle (CS, NADP-ICD, MDH) and its associated anaplerotic routes (PEPC, NADP-ME) were also enhanced in RA (Fig. 1).

**Table 1:** Wheat plant biomass, leaf chlorophyll and root content of N, C, NH_4_^+^, amino acid and soluble protein from wheat plants grown under nitrate or ammonium nutrition. Values represent mean ± SE (n = 6; for biomass n = 18). Asterisk (*) indicates significant differences between N nutritions (p < 0.05).

	Nitrate		Ammonium
Shoot biomass (g DW plant^−1^)	2.15 ± 0.17	*	1.56 ± 0.09
Root biomass (g DW plant^−1^)	0.93 ± 0.09	*	0.40 ± 0.01
Chlorophyll (mg g^−1^ DW)	16.28 ± 0.31	*	17.42 ± 0.20
**Root**			
N content (%)	3.71 ± 0.13		4.04 ± 0.21
C content (%)	37.50 ± 1.22	*	42.81 ± 0.28
NH_4_^+^ (µmol g^−1^ DW)	17.50 ± 4.48	*	99.87 ± 25.28
Amino acids (µmol g^−1^ DW)	51.60 ± 10.65	*	227.98 ± 8.68
Protein (mg protein g^−1^ DW)	63.78 ± 6.03	*	88.42 ± 5.43

### Isotopic labelling of roots with ^15^NH_4_^+^ or ^15^NO_3_^−^

**Figure 2:**
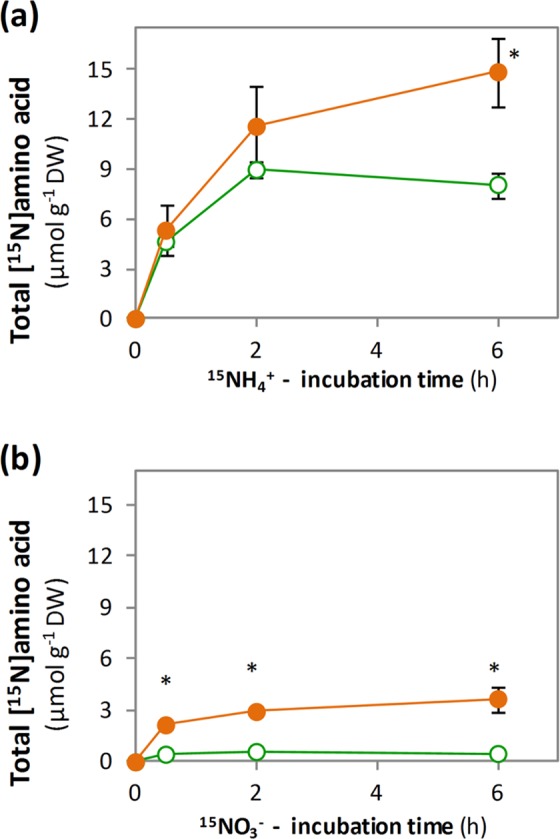
Total labelled amino acid accumulation after (**a**) ^15^NH_4_^+^ or (**b**) ^15^NO_3_^−^ incubation of roots of wheat plants grown under nitrate (RN; green open circle) or ammonium (RA; orange closed circle) nutrition. Values represent mean ± SE (n = 3). Asterisk (*) indicates significant differences between N nutritions (p < 0.05).

To dig into the effect of the plant’s adaptation to the N source in the *in vivo* biosynthesis of amino acids, ^15^N isotopic labelling analyses were performed in RN and RA. The label was supplied as 10 mM of ^15^NH_4_^+^ and also as ^15^NO_3_^−^ up to a maximum of 6 h to roots adapted to each N source. The incubation of the roots with ^15^NH_4_^+^ allowed the incorporation of the ^15^N label into amino acids in both RA and RN (Fig. 2a). In RA-^15^NH_4_^+^ the total amount of [^15^N]amino acids increased along with the time of incubation up to 14.9 µmol g^−1^ DW. In contrast, the content of [^15^N]amino acids in RN-^15^NH_4_^+^ only reached a maximum value of *ca*. 9 µmol g^−1^ DW at 2 h of incubation. When roots were incubated with ^15^NO_3_^−^ (Fig. 2b), the synthesis of [^15^N]amino acids was significantly lower respect to the incubation with ^15^NH_4_^+^. Importantly, total [^15^N]amino acids were still higher in RA compared to RN. In RA-^15^NO_3_^−^ the maximum content was 3.7 µmol g^−1^ DW at 6 h and in RN-^15^NO_3_^−^ the maximum content was 0.6 µmol g^−1^ DW at 2 h of incubation.

The ^15^N enrichment values in individual amino acids, calculated as the percentage (%) of each labelled amino acid with respect to the total amino acid, (Supplementary Table S1) were lower in RA-^15^NH_4_^+^ as a consequence of the higher initial pools of those amino acids (Supplementary Fig. S2). From the beginning of the incubation with ^15^NH_4_^+^, the most enriched amino acid was [^15^N]Gln under both N nutritions, followed by [^15^N]Glu, [^15^N]Asp and [^15^N]Ala (Supplementary Table S1). Attending to the distribution of the label into the amides ([^15^N]Asn and [^15^N]Gln), the enrichment of each N atom (M + 1 or M + 2) was also calculated separately.  At least a third of both total [^15^N]Gln and [^15^N]Asn had incorporated the label into both N atoms (Supplementary Fig. S3) in both RA-^15^NH_4_^+^ and RN-^15^NH_4_^+^, evidencing the use of already labelled precursors ([^15^N]Glu and [^15^N]Asp, respectively) for its synthesis. The ^15^N enrichment values after the incubation of the root with ^15^NO_3_^−^ were much lower for every amino acid compared to their respective enrichment values when ^15^NH_4_^+^ was supplied (Supplementary Table S2). The labelling pattern in the amides, in contrast, did not show differences between both incubation assays (Supplementary Fig. S4).

**Figure 3:**
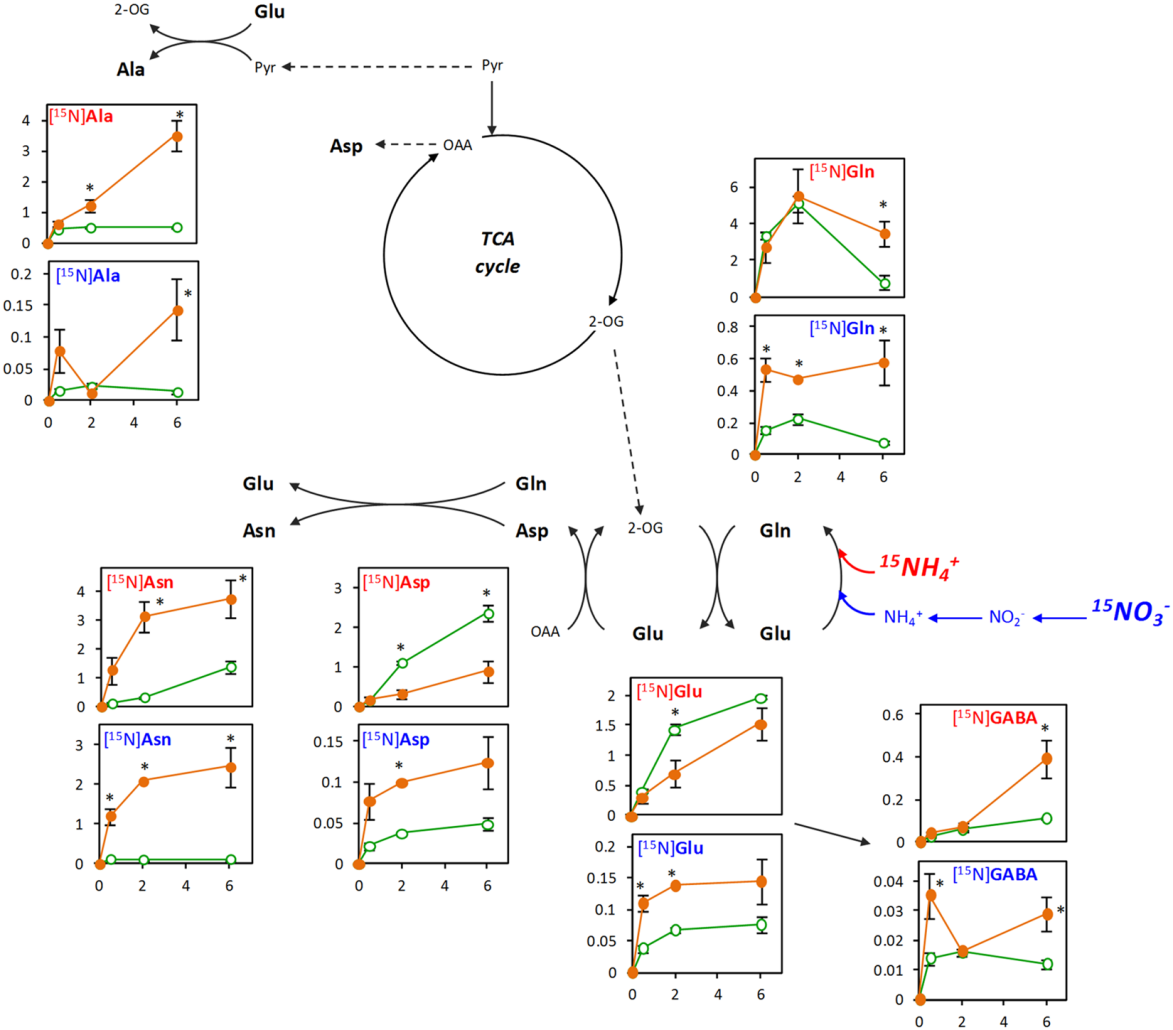
Major [^15^N]amino acid contents (µmol g^−1^ DW) after ^15^NH_4_^+^-incubation (red) or ^15^NO_3_^−^ -incubation (blue) of roots of wheat plants grown under nitrate (RN; green open circle) or ammonium (RA; orange closed circle) nutrition. X-axis indicates the incubation times: 0.5, 2 and 6 h. Values represent mean ± SE (n = 3). Asterisk (*) indicates significant differences between N nutritions (p < 0.05).

The content of the major [^15^N]amino amino acids ([^15^N]Asn, [^15^N]Gln, [^15^N]Ala, [^15^N]Glu, [^15^N]Asp and [^15^N]GABA) after incubation of wheat roots with ^15^NH_4_^+^ or ^15^NO_3_^−^ are shown in Fig. 3. In roots incubated with ^15^NH_4_^+^ (in red) [^15^N]Gln was the most abundant amino acid during the first 2 h under both N nutritions, reaching a maximum of *ca*. 5 µmol g^−1^ DW and then decreasing for both nutritions. In contrast, the content of [^15^N]Glu, as well as the content of the most abundant Glu-derived amino acids, continuously increased (Fig. 3). From the second hour, the most abundant amino acid was [^15^N]Asp for RN-^15^NH_4_^+^ and [^15^N]Asn and [^15^N]Ala for RA-^15^NH_4_^+^ (Fig. 3). Regarding less abundant amino acids, the content of [^15^N]Ser, [^15^N]Val and [^15^N]Ile also continuously increased along the whole incubation period in RA-^15^NH_4_^+^ and RN-^15^NH_4_^+^ (Supplementary Fig. S5). In roots incubated with ^15^NO_3_^−^ (in blue), the content of major [^15^N]amino acids was higher in RA-^15^NO_3_^−^ compared to RN-^15^NO_3_^−^ from the beginning of the incubation period, especially for [^15^N]Asn (Fig. 3). The contents of minor amino acids were also higher in RA-^15^NO_3_^−^, except for [^15^N]Pro and [^15^N]Phe (Supplementary Fig. S6).

**Figure 4:**
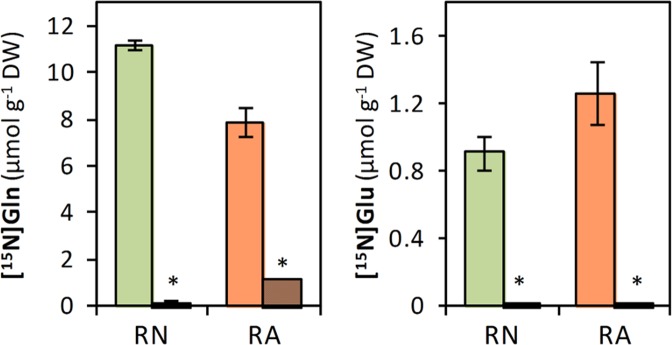
Contents of [^15^N]Glu and [^15^N]Gln in wheat roots grown under nitrate (RN; green) or ammonium (RA; orange) nutrition incubated with ^15^NH_4_^+^ for 30 minutes in absence (plain bars) or in presence (striped bars) of inhibitors methionine sulfoximine (MSX) and azaserine (AZA). Values represent mean ± SE (n = 3). Asterisk (*) indicates significant differences between N nutritions (p < 0.05).

As previously mentioned, GDH enzyme activity was highly induced in RA (Fig. 1). GDH is a reversible enzyme that *in vitro* can both assimilate NH_4_^+^ to form Glu or deaminate Glu to form 2-OG. To ascertain the *in vivo* role of GDH, its capacity to incorporate ^15^NH_4_^+^ to form [^15^N]Glu was evaluated. As shown in Fig. 4 and Supplementary S7, the incubation of root segments with GS/GOGAT pathway inhibitors MSX and AZA highly reduced or even abolished the incorporation of ^15^NH_4_^+^ into amino acids. Importantly, the newly synthesized [^15^N]Glu was almost undetectable (Fig. 4).

### Isotopic labelling of roots with [^13^C]Pyr

**Figure 5:**
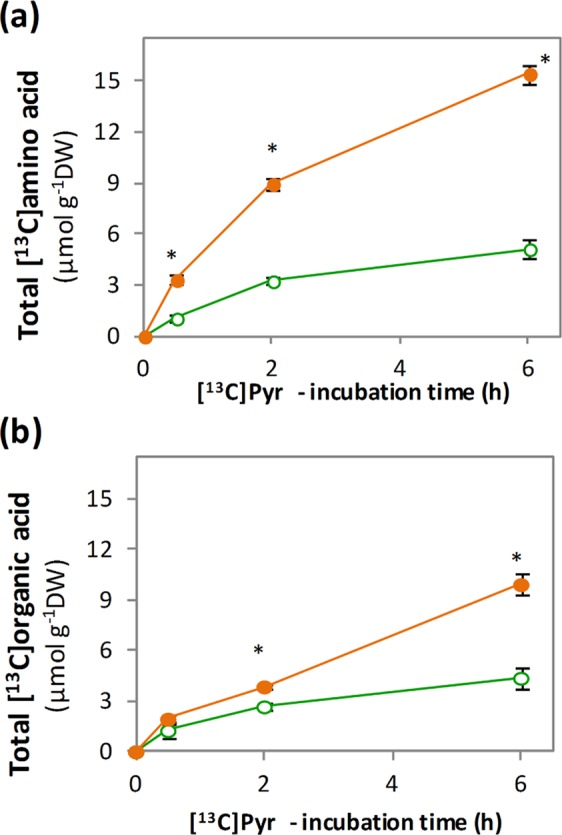
Total labelled (**a**) amino acid and (**b**) organic acid accumulation after [^13^C]Pyr-incubation of roots of wheat plants grown under nitrate (RN; green open circle) or ammonium (RA; orange closed circle) nutrition. Values represent mean ± SE (n = 3). Asterisk (*) indicates significant differences between N nutritions (p < 0.05).

To gain insight on how TCA metabolic flux is modulated *in vivo* in roots adapted to nitrate or ammonium nutrition, RA and RN were supplied with 10 mM [^13^C]Pyr, plus the corresponding N source (10 mM of unlabelled NH_4_^+^ or NO_3_^−^), and ^13^C incorporation into organic acids and amino acids was followed. The formation of [^13^C]amino acids showed a logarithmic increasing trend under both N nutritions; however, total [^13^C]amino acid content was higher in RA, reaching a maximum of 15.4 µmol g^−1^ DW at 6 h compared to 5.1 µmol g^−1^ DW in RN (Fig. 5). Regarding total [^13^C]organic acids, they accumulated following a linear regression, which increased from *ca*. 2 µmol g^−1^ DW at 30 min to *ca*. 10 µmol g^−1^ DW at 6 h in RA, while in RN only reached a maximum of 4.3 µmol g^−1^ DW at 6 h (Fig. 5).

Observing [^13^C]molecules individually (Supplementary Table S3), around 80% of the [^13^C]Pyr was labelled in both RN and RA during the whole incubation period, whilst the ^13^C enrichment for the rest of organic acids increased with time. The most abundant amino acids also showed an increasing enrichment pattern over the incubation period. Looking deeper into the labelling pattern of the organic acids (Supplementary Table S4), practically all the [^13^C]Pyr was labelled in its three carbons (m + 3) during the whole incubation period with 10 mM [^13^C]Pyr; while the rest of [^13^C]organic acids were, in general, more labelled in two carbons (m + 2). By the end of the incubation period, the second most labelled mass peak was m + 3 (molecule labelled in three C) for [^13^C]Citrate and [^13^C]Isocitrate under both N nutritions. In contrast, for [^13^C]Succinate, [^13^C]Fumarate and [^13^C]Malate the second most labelled mass peak was m + 1 (molecule labelled only in one C). In the case of [^13^C]2-OG, the label distribution differed between N nutrition, with the second most abundant peak being m + 1 in RN and m + 3 in RA. The major [^13^C]amino acids were again [^13^C]Ala, [^13^C]Glu, [^13^C]Gln, [^13^C]Asp, [^13^C]Asn and [^13^C]GABA (Supplementary Table S4). Since Ala directly derives from Pyr, most of [^13^C]Ala present in the roots at 30 min was labelled in its three carbons (m + 3) regardless the previous N nutrition. In contrast, the other five amino acids were mostly labelled in two carbons (m + 2); followed by one-carbon labelling (m + 1) for [^13^C]Asp, [^13^C]GABA and [^13^C]Asn and by three-carbon labelling (m + 3) for [^13^C]Glu and [^13^C]Gln (Supplementary Table S4).

**Figure 6:**
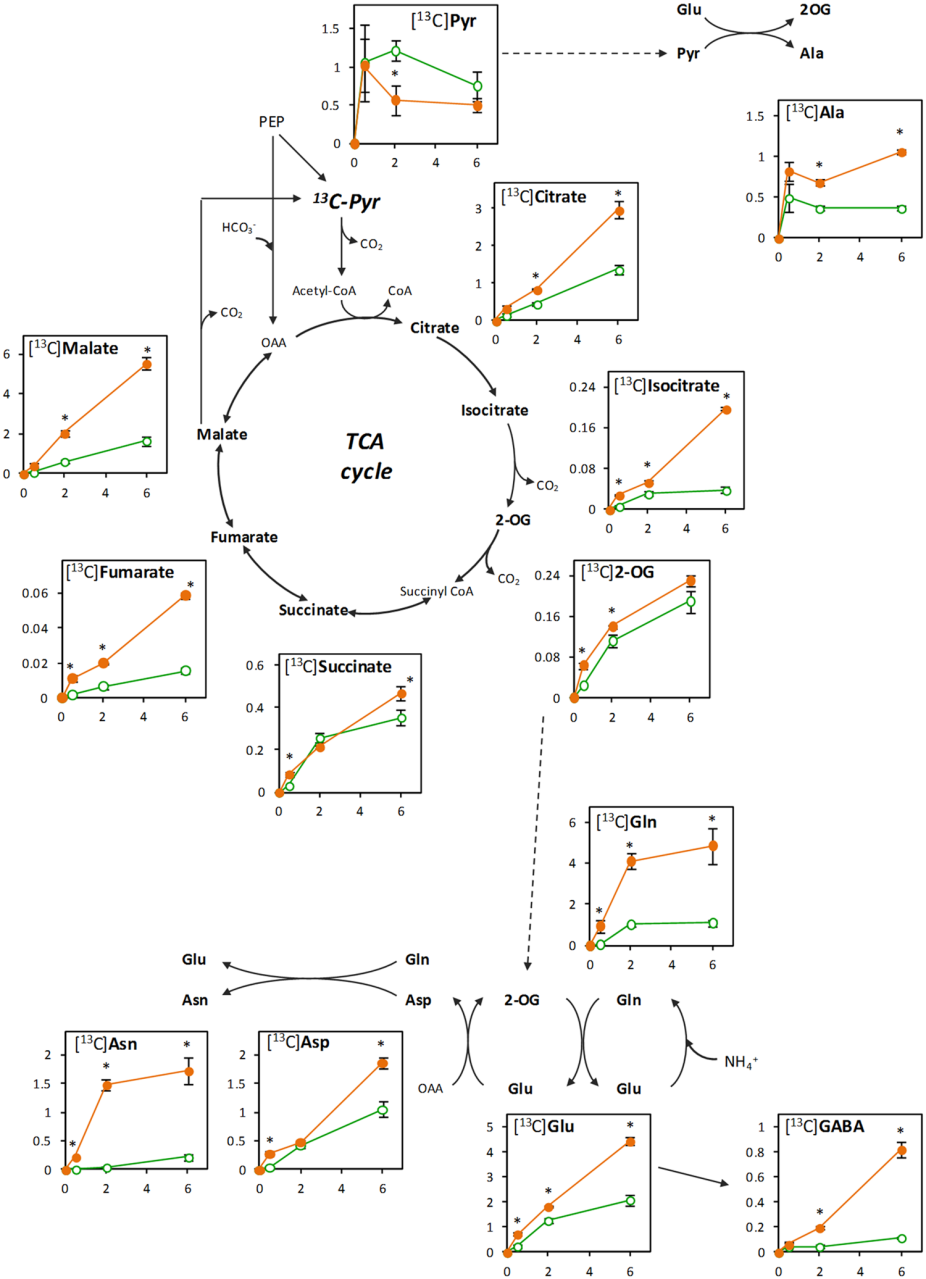
Major [^15^N]amino acid and [^13^C]organic acid contents (µmol g^−1^ DW) after [^13^C]Pyr-incubation of roots of wheat plants grown under nitrate (RN; green open circle) or ammonium (RA; orange closed circle) nutrition. X-axis indicates the incubation times: 0, 0.5, 2 and 6 h. Values represent mean ± SE (n = 3). Asterisk (*) indicates significant differences between N nutritions (p < 0.05).

Concerning the content of individual molecules in RA and RN (Fig. 6), the [^13^C]Pyr itself was the most abundant [^13^C]organic acid in the first 30 min, although its content decreased along time. The other [^13^C]organic acids, on the contrary, accumulated linearly, notably [^13^C]Malate, [^13^C]Citrate, [^13^C]Isocitrate and [^13^C]Fumarate, reaching higher contents in RA compared to RN. Besides, ammonium nutrition also led to higher accumulation of major [^13^C]amino acids in roots incubated with [^13^C]Pyr compared to nitrate nutrition (Fig. 6). In RA, [^13^C]Gln and [^13^C]Asn strongly increased in the first 2 h and then stabilized, whilst [^13^C]Glu, [^13^C]Asp and [^13^C]GABA continued increasing linearly until the end of the incubation. Attending to less abundant [^15^N]amino amino acids, [^13^C]Ser, [^13^C]His, [^13^C]Met, [^13^C]Thr, [^13^C]Ile, [^13^C]Leu and [^13^C]Gly contents were also higher in RA compared to RN (Supplementary Fig. S8).

## Discussion

### The induction of root metabolic pathways for amino acid synthesis is essential to face ammonium stress

Ammonium nutrition entails a growth constraint in most plants including cereals such as barley^32^ and wheat^16,31,33^, as also observed in the present study, mainly as a consequence of reduced root biomass (Table 1; Supplementary Fig. S1). The wheat root acts as a physiological barrier that accomplished two main strategies to face ammonium stress: to increase NH_4_^+^ assimilation into amino acids and protein; and to accumulate the excess free NH_4_^+^ (Table 1). The enhanced assimilation of NH_4_^+^ demands a consequent C skeleton supply^25,33^. Thereby the induced activities related to the TCA cycle, together with GS/GOGAT pathway, would be in the charge of such an enhanced NH_4_^+^ assimilation in RA (Fig. 2), as also previously observed in different species^15,16^. Particularly, PEPC activity has been shown to increase under ammonium nutrition^18,26^ and to correlate with GS activity^34^. The investment of C skeletons in the synthesis of amino acids is further reflected by organic acids content decrease in RA compared to RN (Supplementary Fig. S2).

In order to optimize the storage of N compounds in the cell, the main amino acids accumulated in RA were those with higher N:C ratio (Asn and Gln; Supplementary Fig. S2). Asn stands out as a long-distance N transporter on whole plant basis^35^ and it is usually the main amino acid accumulated in monocots under ammonium nutrition^16,36^. Therefore, evidencing that AS may act as a complement of GS/GOGAT pathway in NH_4_^+^ detoxification^37^. Additionally, the observed induction of GDH activity (Fig. 1), due to its reversible nature, may be contributing to the synthesis of Glu or 2-OG. Nonetheless, *in vitro* determined activities do not always reflect the dynamic of metabolic fluxes and thus, an *in vivo* isotopic-labelling approach was engaged to provide a dynamic depiction of root N assimilation and TCA cycle tuning in response to N nutrition.

### *In vivo*^15^NH_4_^+^ labelling evidences the enhanced ammonium assimilation capacity of ammonium-adapted roots through GS/GOGAT cycle

The analysis of [^15^N]amino acids enrichment allowed tracking the *in vivo* course of ^15^NH_4_^+^ provided to wheat roots adapted to grow under different N sources. Importantly, the equal ^15^N label incorporation into amino acids during the first 30 minutes (Figs 2 and 3) denotes a similar N flux in the early stage of the incubation regardless the plant’s previous nutrition. This suggests that the supply of ^15^NH_4_^+^ provoked a metabolic switch in RN-^15^NH_4_^+^ prompting a rapid induction of ammonium assimilation to the levels reported in RA-^15^NH_4_^+^.

The label from ^15^NH_4_^+^ was firstly incorporated into [^15^N]Gln in both RA-^15^NH_4_^+^ and RN-^15^NH_4_^+^ as it was the most enriched amino acid (Supplementary Table S1); thus, highlighting the role of GS as the main ammonium assimilating enzyme in wheat. Besides, the increase in [^15^N]Glu together with the decrease of [^15^N]Gln along time (Fig. 3) indicates that GOGAT is working in tandem with GS. Glu is necessary to sustain not only the synthesis of [^15^N]Gln but is also diverted to the synthesis of other amino acids via transamination, such as [^15^N]Ala or [^15^N]Asp (Table 2; Fig. 3). In RA, the assimilated ^15^NH_4_^+^ was ultimately diverted to [^15^N]Asn, the major storage amino acid in wheat plants (Supplementary Fig. S2). Interestingly, the formation of [^15^N]GABA, that would be sinthetized by decarboxylation via glutamate decarboxylase (GAD) activity, also followed an increasing trend in RA-^15^NH_4_^+^ (Fig. 3). The induction of GABA production is common under stress conditions^38^ and was shown to contribute to the alleviation of ammonium toxicity in rice^39^. Therefore, its synthesis could be an indicator of the stressful situation undergone in response to high NH_4_^+^ contents in wheat roots. Alternatively, GABA could also take part in the GABA shunt, being transaminated with Pyr by GABA transaminase (GABA-T), producing Ala and succinate^40^.

Additionally, [^15^N]Glu could be also converted to 2-OG by GDH. Indeed, one of the common responses of ammonium nutrition is the induction of GDH activity, as observed in Arabidopsis^9^ or wheat^16,41^, which is also reported in the present work (Fig. 1). *In vitro* GDH catalyses both the reductive amination of 2-OG and the oxidative deamination of Glu but *in vivo* its role remains controversial. Studies using tobacco overexpressing GDH isoforms^42^ and Arabidopsis *gdh* mutants^43,44^ demonstrated *in vivo* that GDH central role is Glu deamination. Alternatively, it has also been proposed that under certain conditions GDH could be participating in the direct assimilation of NH_4_^+^, such as during stressful situations where NH_4_^+^ is accumulated^45,46^. In this line, very recently, *in vivo* GDH aminating activity was observed in tomato roots exposed to excess ^15^NH_4_^+^ provision^47^. Similarly, Ferraro *et al*.^48^, using *GDH* knock down tomato lines, also suggested in fruits that GDH could be working *in vivo* in its aminating sense. In the present work, in line with the works advocating for GDH role in Glu deamination, the nearly absence of ^15^N label incorporation into [^15^N]Glu when GS/GOGAT pathway is inhibited and the slight incorporation of the label into other amino acids (Figs 4, S7) led us to discard a significant participation of GDH in the *in vivo* assimilation of ^15^NH_4_^+^ in wheat roots. Therefore, the enhanced GDH activity observed in RA (Fig. 1) could mean increased 2-OG provision in wheat roots, essential for the detoxification of NH_4_^+^ into Gln via GS/GOGAT pathway or into Asp and Asn via OAA formation in the TCA cycle. The results presented in this paper together with the available literature suggest that *in vivo* GDH role would be different depending on the plant organ, on plant growth and environmental conditions and, importantly, also on the plant species.

Importantly, although roots adapted to both N nutritions showed a similar immediate response to ^15^NH_4_^+^ supply, RA-^15^NH_4_^+^ was able to maintain higher ^15^NH_4_incorporation rates along time, as illustrated by the raised accumulation of ^15^N label into Asn and Ala at 6 h (Fig. 3). Thereby, this result demonstrates that the activation of enzymes responsible for ammonium assimilation in plants adapted to an ammonium-based nutrition conferred the root improved capacity to assimilate ^15^NH_4_^+^ into amino acids. However, the diversion of plant’s C resources to ammonium detoxification may be a trade-off for plant growth. Thus, highlighting that the fine regulation of C:N balance is key for the plant performance under ammonium nutrition.

### ^15^NO_3_^−^ labelling underscores the relevance of ammonium assimilation machinery for efficient nitrate incorporation into amino acids

Wheat root metabolic fluxes were also determined in response to ^15^NO_3_^−^ supply, among others to evaluate whether RN-^15^NO_3_^−^, with a more active NR enzyme (Fig. 1), was able to use nitrate more efficiently than RA-^15^NO_3_^−^. The reduction of NO_3_^−^ is highly dependent of adequate reductant (Fd and NAD(P)H) availability, and in fact, NR activity has long been considered to be the rate-limiting step in most plants^3^. In this work, the formation of [^15^N]amino acids from ^15^NO_3_^−^ was much lower compared with that of direct ^15^NH_4_^+^ supply both in RN and RA (Fig. 3). Therefore, these results reinforce that the energetic cost of nitrate primary assimilation supposes a bottleneck that slows down the use of ^15^NO_3_^−^ as N source.

Interestingly, RA-^15^NO_3_^−^showed again a higher capacity to synthesize [^15^N]amino acids compared to RN-^15^NO_3_^−^ (Figs 2b, 3). So, the fact that the roots of plants previously adapted to an exclusive ammonium-based nutrition favoured ^15^NO_3_^−^ assimilation compared to plants adapted to nitrate nutrition would be indicating that the pre-activation of nitrate uptake and reduction machinery would not be an advantage to use ^15^NO_3_^−^ more efficiently. Alternatively, RA-^15^NO^3−^ clearly benefits from the previously induced GS/GOGAT activities and more active TCA cycle (Fig. 1) that provide C and metabolic energy to efficiently reduce and assimilate ^15^NO_3_^−^ into amino acids, particularly [^15^N]Asn (Fig. 3).

### [^13^C]Pyr highlights the flexibility of TCA cycle to meet de demand of C skeletons for efficient ammonium detoxification

**Figure 7:**
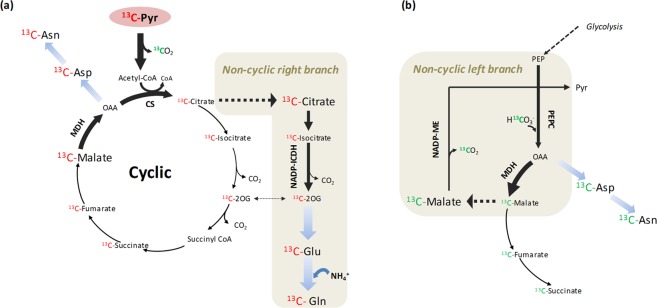
TCA cycle flux modes inferred from the [^13^C]Pyr-labelling of wheat roots preadapted to ammonium nutrition. (**a**) Cyclic respiratory cycle and non-cyclic right branch for 2-OG (2-oxoglutarate) provision. The principal amino acid formation is also indicated. (**b**) Non-cyclic left branch with the participation of anaplerotic enzymes. The reassimilation of ^13^C label coming from [^13^C]Pyr decarboxilation step is indicated in green. TCA cycle-related enzyme activities that were determined in wheat roots are indicated (CS, MDH, NADP-ICDH, NADP-ME, and PEPC).

In order to explore the rearrangement of the TCA cycle in response to the N source *in vivo*, [^13^C]Pyr was supplied to wheat roots. [^13^C]Pyr is decarboxylated by pyruvate dehydrogenase and thus, the ^13^C label enters into TCA cycle as a two-carbon acetyl CoA molecule. In one ‘turn’ of the cycle, two carbons are lost as CO_2_ but the entering labelled acetyl carbons remain present in the recycled OAA molecule^49^. In wheat roots, the incorporation of [^13^C]Pyr into TCA cycle led to the subsequent enrichment of the intermediaries, starting from [^13^C]Citrate and reaching [^13^C]Malate (Supplementary Table S3). Moreover, all the [^13^C]organic acids from the cycle showed to be labelled mostly in two of their C atoms (Supplementary Table S4), confirming that the supply of [^13^C]Pyr allowed the fuelling of ^13^C along the complete TCA cycle (Figs. 6 and 7a).

Although the initial uptake of [^13^C]Pyr was equal for both N sources, the higher consumption of [^13^C]Pyr in RA (Fig. 6) agrees with the higher synthesis of both total [^13^C]organic acids and [^13^C]amino acids (Fig. 5), evidencing that wheat plant adaptation to ammonium nutrition induces the acceleration of C metabolism through TCA cycle. Such response is principally illustrated by the enhanced [^13^C]Citrate accumulation in RA (Fig. 6), sustained by the remarkable CS activity (Fig. 2). Citrate, as one of main C reservoirs in the cell, will assure the functioning of the respiratory cycle^22,50,51^. Indeed, the labelling study carried out by Gauthier *et al*.^52^ showed that N assimilation in illuminated *Brassica napus* leaves was principally maintained by the citrate and malate stored during the night period. On the other hand, despite the higher ICDH activity in RA (Fig. 2), the content and accumulation dynamic of [^13^C]2-OG was almost equal in both RA and RN due to its extremely rapid use for the synthesis of [^13^C]Glu and [^13^C]Gln (Fig. 6). Low levels of 2-OG are usually detected in the cell when compared to other organic acid (Supplementary Fig. S2) because it is rapidly incorporated into diverse N-containing molecules. Indeed, the predominance of [^13^C]amino acids labelled in two of their carbons (Table S4) underlined that [^13^C]2-OG was the principal C skeleton donor from [^13^C]Pyr, and therefore essential in detoxification of high ammonium contents in wheat root. Likewise, part of the ^13^C flux continued alongside the TCA cycle leading to high [^13^C]Malate contents, especially in RA (Fig. 6), that lead to the improved formation of two-carbon labelled [^13^C]Asp and [^13^C]Asn via OAA (Table 3, Fig. 7a). 2-OG has been also suggested to function as a signalling molecule, among others, for the regulation of primary metabolism in eukaryotic algae and cyanobacteria^53,54^. The equal levels reported in RA and RN may suggest a cell control of 2-OG levels in line with its potential role in the control of C/N metabolic interactions.

The divert of [^13^C]2-OG for the enhanced synthesis of [^13^C]Glu and [^13^C]Gln under ammonium nutrition leads to an open TCA flux mode (non-cyclic right branch, Fig. 7a) that requires the replenishment of the intermediaries of the cycle by the anaplerotic routes. Several alternative flux modes were proposed in which the initial substrate of the cycle will be PEP instead of being Pyr^22,49,55^. In this line, in the late period of the incubation with [^13^C]Pyr, a substantial part of [^13^C]Succinate, [^13^C]Fumarate and [^13^C]Malate showed to be labelled only in one carbon (Table 4). The most plausible explanation is that PEPC will be reassimilating the ^13^CO_2_ from [^13^C]Pyr decarboxylation into one-carbon labelled OAA that will be fuelling the left branch of the cycle. This reassimilation was also observed in leaves after the provision of ^13^C labelled substrates ([^13^C]Pyr, [^13^C]Glucose, or ^13^CO_2_)^51,56^. Based on these results, an open flux model is proposed for ammonium-adapted wheat roots (Fig. 7b) where the TCA-related anaplerotic enzymes accomplish a pivotal role. In accordance with this model, [^13^C]OAA will be diverted both to the synthesis of one-carbon labelled [^13^C]Asp by AAT or one-carbon labelled [^13^C]Malate through the induced reversible activity of MDH (Fig. 2). Finally, [^13^C]Malate can be decarboxylated by NADP-ME in the cytosol to produce unlabelled Pyr, which will enter again into the mitochondrial TCA cycle (Fig. 7b). This highlights the dual role of malate, together with citrate, in the TCA cycle to produce energy or carbon skeletons. Therefore, the coordinated operation of PEPC, MDH and ME in different organelles provides TCA cycle with the flexibility to function in an open-mode according to the metabolic demands of the cell^22,29,57^.

In conclusion, *in vivo* metabolic flux analysis evidenced that the enhanced ammonium assimilation machinery together with the increased C flux through non-cyclic TCA pathways are essential to sustain the needed amino acid synthesis for the detoxification of the NH_4_^+^. ^15^N labelling together with inhibitor assays asserted that NH_4_^+^ is exclusively assimilated via GS/GOGAT in wheat roots, discarding the participation of GDH. Besides, the adaptation of the root to ammonium nutrition is an advantage that promotes a more efficient assimilation of the supplied N, both in form of NO_3_^−^ or NH_4_^+^. Such response would mean a greater use of the different N sources available in the soil by ammonium-adapted plants that could be a key strategy to increase their assimilation NUE. Thus, the control of the nitrogen source availability, notably through promoting the use of ammonium-based fertilizers, merits to take a bigger place in fertilization management strategies, since it could be of interest to maximize NUE by the plant and thus to reduce the impact of nitrogen fertilization on the environment.

## Materials and Methods

### Growth conditions

Seeds of wheat (*Triticum aestivum* L. var. Cezanne) were germinated in trays filled with perlite:vermiculite (1:1; v-v) mixture and watered with deionised water. Trays were kept during 4 days in the dark at 4 °C for vernalization and transferred into a growth chamber with 14 h light photoperiod (light intensity of 450 μmol m^−2^ s^−1^). Temperature was 23 °C in the light period and 18 °C in the dark period, with a relative humidity of 60% and 70%, respectively. Two weeks after sowing, 12 seedlings were transferred to 13 L hydroponic tanks with continuous aeration. Six tanks were set up per condition. The nutrient solution contained macronutrients (1.15 mM K_2_HPO_4_, 0.85 mM MgSO_4_, 0.7 mM CaSO_4_, 2.68 mM KCl, 0.5 mM CaCO_3_, 0.07 mM NaFeEDTA) and micronutrients (16.5 μM Na_2_MoO_4_, 3.7 μM FeCl_3_, 3.5 μM ZnSO_4_, 16.2 μM H_3_BO_3_, 0.47 μM MnSO_4_, 0.12 μM CuSO_4_, 0.21 μM AlCl_3_, 0.126 μM NiCl_2_ and 0.06 μM KI). The N source was 5 mM Ca(NO_3_)_2_ for nitrate-fed plants and 5 mM SO_4_(NH_4_)_2_ for ammonium-fed ones. To appropriately compare both N sources, nitrate-fed plants were supplied with 5 mM CaSO_4_ to equilibrate the sulphate supplied together with the ammonium. The nutrient solution was changed weekly and pH maintained around 6. After six weeks three plants per tank were individually harvested and dried in an oven at 80 °C for 72 h for biomass determination. Other three plants per tank were pooled, snap frozen in liquid nitrogen, ground to powder and stored at −80 °C for metabolite and enzymatic analyses. The resting plants were used for labelling assays. Fresh to dry mass ratio was calculated and used to refer metabolite contents and enzymatic activities on dry weight (DW) basis.

### Determination of N, C, free ammonium and chlorophyll

Total N and C content were quantified from dry material by elemental analyser Flash EA1112 (Thermo Fisher Scientific Inc., Waltham, MA, USA).

Free ammonium content was determined using the colorimetric method based on the phenol hypochlorite assay (Berthelot reaction) in aqueous extracts obtained by the homogenization of 25 mg of frozen material with 500 µL of ultrapure water. The homogenates were incubated 5 min at 80 °C and centrifuged at 4000 *g* and 4 °C. Chlorophyll content was determined following the protocol established by Arnon *et al*.^58^. To do so, 50 mg of frozen leaf tissue was extracted in 1 mL of 80% aqueous acetone, the homogenates were centrifuged at 12 000 *g* and 4 °C and the absorbance from the supernatant measured at 645 and 663 nm.

### Protein extraction and enzyme activities determination

Protein was extracted from 150 mg of frozen root powder with 1.5 mL of extraction buffer described in Sarasketa *et al*.^9^. For soluble protein quantification a Bradford based dye-binding assay (Bio-Rad, Hercules, CA, USA) was employed, using bovine serum albumin as standard.

Every enzyme was determined with a 96-well microplate reader (BioTek Instruments). For every enzyme except glutamine synthetase (GS), nitrate reductase (NR) and citrate synthase (CS), 20 µL of extract were incubated during 20 min at 30 °C with 280 µL of reaction buffer and the evolution of NAD(P)H monitored at 340 nm. For NADH-dependent glutamate dehydrogenase (GDH) NADH-dependent glutamate synthase (GOGAT), phosphoenolpyruvate carboxylase (PEPC), NADP-dependent malic enzyme (NADP-ME), malate dehydrogenase (MDH) and NADP-dependent isocitrate dehydrogenase (ICDH) the reaction buffers are described in Sarasketa *et al*.^9^. For aspartate aminotransferase (AAT) activity, the reaction buffer contained 50 mM MBM (pH 8), 0.2 mM NADH, 10 mM aspartic acid, 1 mM 2-OG, 3 mM pyridoxal phosphate and 3.6 U of MDH mL^−1^. GS activity was determined following the formation of γ-glutamylmonohydroxamate (γ-GHM) at 540 nm and NR activity following the formation of KNO_2_ at 546 nm as described in Sarasketa *et al*.^59^. For CS enzyme activity, no DTT was added to the extraction buffer described above. CS activity was measured in extracts 20 µL of extract incubated during 20 min at 30 °C with 280 µL of reaction buffer described in Srere *et al*.^60^ and the formation of 2-nitro-5-thiobenzoic acid (TNB) was monitored at 412 nm.

### Isotopic labelling

Six plants per tank were harvested and the roots from two tanks were pooled as a sole sample. The roots were washed three times in deionised water and were cut in 3 cm long segments. Root segments (1 g) were pre-incubated during 30 min in 10 mL buffer solution (10 mM MES + KOH, pH 6.5), in order to acclimate the root pieces to the medium.

Labelling was provided by the addition of 5 mM (^15^NH_4_)_2_SO_4_ (98 atom % ^15^N, Ref. 299286, Sigma-Aldrich), 5 mM (^15^NO_3_)_2_Ca (98 atom % ^15^N, Ref. 488410, Sigma-Aldrich) or 10 mM sodium pyruvate−^13^C_3_ ([^13^C]Pyr; 99 atom % ^15^N, Ref. 490717, Sigma-Aldrich). Each labelling was performed in the roots coming from plants grown during six weeks under nitrate nutrition (RN) or under ammonium nutrition (RA). In parallel, control root samples were prepared by adding the equivalent unlabelled substrates. For the incubation with [^13^C]Pyr, N source was also supplied to RN or RA as 5 mM (NO_3_^−^)_2_Ca or (NH_4_^+^)_2_SO_4_, respectively. For every condition and time point three independent samples were analyzed. Root segments were collected at 0, 0.5, 2 and 6 hours after the substrates addition, washed three times with buffer, immediately frozen in liquid nitrogen and stored at −80 °C until extraction for GC-TOF-MS analysis.

As a complementary experiment, in order to inhibit the GS/GOGAT enzyme activities, RN and RA root segments were pre-incubated during 1 hour with 2 mM L-methionine sulfoximine (MSX, Ref. M5379, Sigma-Aldrich) and 1.75 mM Azaserine (AZA, Ref. A4142, Sigma-Aldrich) in the buffer solution; afterwards, roots were incubated during 30 min with 5 mM (^15^NH_4_)_2_SO_4_.

### Extraction of amino acids and organic acids

Root samples were freeze dried, and 20 mg of dry material was incubated with 1.8 ml of methanol:chloroform:H_2_O (1:2.5:1 v:v:v) in a rotary shaker for 30 min at 4 °C. After a 5 min centrifugation at 8000 *g* and 4 °C, the aqueous phase was dried in a Savant SpeedVac vacuum concentrator (Thermo Scientific^TM^). The obtained pellets were resuspended in 1000 µl ultra-pure water and filtered (CHROMAFIL® Xtra PA, 20 μm) for amino acid and organic acid determination.

### Quantification of ^15^N labelled amino acids by gas chromatography coupled to mass spectrometry (GC-MS)

Vacuum dried amino acid extracts were resuspended in 50 µl of the derivatizing mixture composed of N-methyl-N-tert-butyldimethylsilyl-trifluoroacetamide (MBDSTFA, Macherey Nagel, France), acetonitrile and triethylamine (15:15:1, v-v:v) and incubated at 95 °C for 30 min in a dry incubator. Alpha aminobutyric acid [α-ABA (IS)] was added as internal standard.

^15^N analyses were carried out by GC-MS (436 GC-MS Scion; Bruker) according to the protocol described in Cukier *et al*.^61^. The quantification of ^15^N enrichment (%) and the amount of each amino acid is based on the determination of the areas of characteristic peaks: The peak of mass M, corresponding to the unlabelled (^14^N) amino acid, and the peaks of mass M + n, corresponding to labelled amino acids with n designing the number of ^15^N atoms integrated either naturally or during the labelling experiment. The ^15^N enrichment (%) in labelled samples was calculated as the ratio of the area (A) of the labelled peak (M + n) to the sum of all the peaks as:

$${}^{15}\,{\rm{N}}\,{\rm{enrichment}}\,( \% )={\rm{A}}({\rm{M}}+{\rm{n}})/[{\rm{A}}({\rm{M}})+{\rm{A}}({\rm{M}}+{\rm{n}})]\ast 100$$

For amino acids with more than one N atom, the enrichment values of each N atom were summed. For example, for glutamine (Gln) three peaks can be observed, an unlabelled peak (M) and two labelled peaks (M + 1) and (M + 2) corresponding to the Gln molecules with one ^15^N atom (singly labelled) and two ^15^N atoms (doubly labelled). So, the ^15^N enrichment (%) was calculated as:

$$[{}^{15}\,{\rm{N}}]\,{\rm{G}}{\rm{l}}{\rm{n}}\,{\rm{e}}{\rm{n}}{\rm{r}}{\rm{i}}{\rm{c}}{\rm{h}}{\rm{m}}{\rm{e}}{\rm{n}}{\rm{t}}\,({\rm{ \% }})=[{\rm{A}}({\rm{M}}+1)+{\rm{A}}({\rm{M}}+2)]/[{\rm{A}}({\rm{M}})+{\rm{A}}({\rm{M}}+1)+{\rm{A}}({\rm{M}}+2)]\ast 100$$

In order to distinguish in labelled samples the proportion of M + n due to natural abundance and that due to the incorporation of the ^15^N during the experiments, a correction factor (cf_n_) is calculated for each unlabelled amino acid as:

$${{\rm{cf}}}_{{\rm{n}}}={\rm{A}}({\rm{M}}+{\rm{n}})/{\rm{A}}({\rm{M}})$$

Standard pure amino acids are used for this analysis. This correction factor is determined experimentally for each fragment ion of each amino acid and will be used to quantify the amount of naturally enriched amino acids in labelled samples as:

$${({\rm{M}}+{\rm{n}})}_{{\rm{n}}{\rm{a}}{\rm{t}}{\rm{u}}{\rm{r}}{\rm{a}}{\rm{l}}}={\rm{A}}\,({\rm{M}})\ast {{\rm{c}}{\rm{f}}}_{{\rm{n}}}$$

This value will be subtracted from the total amount of the labelled amino acid. So, the ^15^N enrichment (%) was finally calculated as:

$$\begin{array}{lll}{}^{15}{\rm{N}}\,{\rm{e}}{\rm{n}}{\rm{r}}{\rm{i}}{\rm{c}}{\rm{h}}{\rm{m}}{\rm{e}}{\rm{n}}{\rm{t}}({\rm{ \% }}) & = & [{\rm{A}}{({\rm{M}}+{\rm{n}})}_{{\rm{m}}{\rm{e}}{\rm{a}}{\rm{s}}{\rm{u}}{\rm{r}}{\rm{e}}{\rm{d}}}-{\rm{A}}{({\rm{M}}+{\rm{n}})}_{{\rm{n}}{\rm{a}}{\rm{t}}{\rm{u}}{\rm{r}}{\rm{a}}{\rm{l}}}]/[{\rm{A}}({\rm{M}})\\  &  & +({\rm{A}}{({\rm{M}}+{\rm{n}})}_{{\rm{m}}{\rm{e}}{\rm{a}}{\rm{s}}{\rm{u}}{\rm{r}}{\rm{e}}{\rm{d}}}-{\rm{A}}{({\rm{M}}+{\rm{n}})}_{{\rm{n}}{\rm{a}}{\rm{t}}{\rm{u}}{\rm{r}}{\rm{a}}{\rm{l}}})]\ast 100\end{array}$$

The total amount of each amino acid is calculated by the sum of the areas of all the fragment ions (unlabelled and labelled). Calibration curves were obtained for each amino acid by using a mixture of commercial pure amino acids. For individual [^15^N]amino acid contents, the ^15^N enrichment (%) value of each amino acid was multiplied by the total content of the respective amino acid. Data are given on a dry weight basis.

### Quantification of ^13^C labelled amino acids and organic acids by GC-MS

For GC-MS analysis, vacuum dried amino acid and organic acid extracts were resuspended in 50 µl of the derivatizing mixture composed of 20 mg/mL methoxyamine hydrochloride in pyridine (Sigma-Aldrich, France) and incubated at 30 °C for 90 min. Then, a second derivatization with 50 µl of N-methyl-N-tert-butyldimethylsilyl-trifluoroacetamide (MBDSTFA, Macherey Nagel, France) was carried out at 70 °C for 30 min. ^13^C analyses were performed by GC-MS (436 GC-MS Scion; Bruker) as described in Cukier *et al*.^61^ for ^15^N analyses. Importantly, for an optimal separation of derivatized ^13^C compounds, the temperature of the oven was regulated according to the following program: 5 min at 70 °C followed by an increase of the temperature at 5 °C min^−1^ until reaching 300 °C and finally 5 min at 300 °C. The ^13^C enrichment (%) calculations were performed in the same way as for [^15^N]amino acids. For [^13^C]amino acid and [^13^C]organic acid contents a maximum of five mass peaks, from M to M + 4, were quantified for each molecule.

### Statistical analysis

Data analyses were carried out using IBM SPSS 20.0 software (IBM Corp.). Statistical analysis of normality and homogeneity of variance were analyzed by Kolmogorov–Smirnov and Levene tests. Statistical differences between nitrate and ammonium nutrition were assessed comparing the mean values by paired t-test. Details of statistical analyses and significance levels are presented in the Fig. legends.

## Supplementary information

Supplementary data

## Data Availability

The datasets generated during and/or analysed during the current study are available from the corresponding author on reasonable request.
