# Supplementary material for: Isotopic labelling reveals the efficient adaptation of wheat root TCA cycle flux modes to match carbon demand under ammonium nutrition
Source: Sci Rep. 2019 Jun 20;9:8925. doi: 10.1038/s41598-019-45393-8 (PMC6586781; doi:10.1038/s41598-019-45393-8)
Supplement: Supplementary file 1 — Supplementary data [file 41598_2019_45393_MOESM1_ESM.pdf]

Isotopic labelling reveals the efficient adaptation of wheat root TCA cycle flux modes to match carbon demand under ammonium nutrition

Izargi Vega-Mas, Caroline Cukier, Inmaculada Coleto, Carmen González-Murua, Anis M. Limami, Mª. Begoña González-Moro, and Daniel Marino

SUPPLEMENTARY DATA

Supplementary Table S1 ^{15}N enrichment of amino acids after $^{15}NH_4^+$ -incubation

Supplementary Table S2 ^{15}N enrichment of amino acids after $^{15}NO_3^-$ -incubation

Supplementary Table S3 ^{13}C enrichment of organic acids and amino acids after [^{13}C]Pyr – incubation

Supplementary Table S4 ^{13}C enrichment of organic acids and amino acids after [^{13}C]Pyr-incubation

Supplementary Figure S1 Picture of wheat plants grown under nitrate or ammonium nutrition

Supplementary Figure S2 Total amino acids and organic acids

Supplementary Figure S3 Label distribution of amides after $^{15}NH_4$-incubation

Supplementary Figure S4 Label distribution of amides after$^{15}NO_3$-incubation

Supplementary Figure S5 Minor [^{15}N]amino acid contents after $^{15}NH_4^+$-incubation

Supplementary Figure S6 Minor [^{15}N]amino acid contents after $^{15}NO_3^-$ -incubation

Supplementary Figure S7 Other [^{15}N]amino acids in presence of inhibitors MSX and AZA

Supplementary Figure S8 Minor [^{15}N]amino acid and [^{13}C]organic acid contents after [^{13}C]Pyr - incubation

Supplementary Table S1. ^{15}N enrichment of amino acids after $^{15}NH_4^+$-incubation of roots of wheat plants grown under nitrate (RN-$^{15}NH_4^+$) or ammonium (RA-$^{15}NH_4^+$) nutrition for 0.5, 2, and 6 h.

	[^{15}N]amino acid enrichment (%)					
	RN-$^{15}NH_4^+$			RA-$^{15}NH_4^+$		
	30 min	2 h	6 h	30 min	2 h	6 h
Ala	9.93 ± 0.63	29.79 ± 2.21	36.57 ± 1.65	5.76 ± 0.17	17.94 ± 0.39	26.54 ± 2.09
Asn	5.47 ± 0.57	9.31 ± 0.79	34.47 ± 5.75	3.58 ± 0.63	6.96 ± 0.67	8.13 ± 0.97
Asp	12.51 ± 0.47	35.15 ± 0.61	37.89 ± 1.32	7.11 ± 0.57	15.85 ± 0.85	18.85 ± 1.71
Gln	73.62 ± 0.93	84.56 ± 0.28	77.27 ± 4.81	23.86 ± 0.67	38.19 ± 4.06	32.62 ± 7.37
Glu	10.43 ± 0.53	36.06 ± 0.54	40.57 ± 1.28	8.41 ± 0.10	23.03 ± 0.50	25.84 ± 1.84
Gly	6.43 ± 0.54	12.39 ± 1.58	13.61 ± 2.28	2.67 ± 0.47	4.79 ± 0.20	5.94 ± 0.33
Ile	3.86 ± 0.38	12.04 ± 0.68	12.50 ± 0.17	2.64 ± 0.84	6.34 ± 0.49	8.83 ± 0.61
Leu	4.15 ± 0.35	13.68 ± 0.45	11.18 ± 0.14	1.85 ± 0.03	6.75 ± 0.25	7.58 ± 0.33
Met	2.15 ± 0.16	6.62 ± 0.19	8.64 ± 0.25	1.53 ± 0.54	2.67 ± 0.70	3.12 ± 0.52
Phe	7.52 ± 0.87	15.00 ± 0.80	9.34 ± 0.73	5.61 ± 0.92	9.92 ± 0.00	8.48 ± 0.00
Pro	7.39 ± 0.26	11.68 ± 0.98	9.04 ± 0.47	5.53 ± 0.00	0.00 ± 0.00	6.07 ± 1.11
Ser	2.12 ± 0.19	5.25 ± 0.11	8.39 ± 0.76	1.38 ± 0.11	3.65 ± 0.13	7.22 ± 0.54
Thr	2.35 ± 0.14	3.56 ± 0.28	2.88 ± 0.18	1.90 ± 0.28	3.28 ± 0.36	2.85 ± 0.03
Tyr	4.62 ± 0.58	10.28 ± 0.89	7.71 ± 0.41	2.76 ± 0.19	6.72 ± 0.24	8.28 ± 0.72
Val	1.48 ± 0.17	4.35 ± 0.12	5.74 ± 0.41	0.68 ± 0.23	2.16 ± 0.41	3.52 ± 0.52
GABA	1.62 ± 0.18	8.27 ± 0.73	31.58 ± 1.13	1.73 ± 0.15	9.14 ± 1.17	19.15 ± 1.45

Values represent mean ± SE (n = 3).

Supplementary Table S2. ^{15}N enrichment of amino acids in wheat roots grown under nitrate (RN-$^{15}NO_3^-$) or ammonium (RA-$^{15}NO_3^-$) nutrition incubated with 10 mM $^{15}NO_3^-$ for 0.5, 2, and 6 h.

	[^{15}N]amino acid enrichment (%)					
	RN-$^{15}NO_3^-$			RA-$^{15}NO_3^-$		
	30 min	2 h	6 h	30 min	2 h	6 h
Ala	0.30 ± 0.07	1.16 ± 0.25	1.16 ± 0.31	0.65 ± 0.27	0.12 ± 0.00	0.90 ± 0.26
Asn	4.08 ± 0.15	4.60 ± 0.40	4.13 ± 0.47	2.38 ± 0.39	2.87 ± 0.00	2.79 ± 0.24
Asp	1.49 ± 0.29	1.31 ± 0.03	1.46 ± 0.13	2.15 ± 0.46	2.19 ± 0.00	1.61 ± 0.43
Gln	4.94 ± 0.25	6.67 ± 0.78	6.62 ± 0.35	3.64 ± 0.02	3.98 ± 0.00	3.47 ± 0.66
Glu	0.69 ± 0.09	1.52 ± 0.05	1.78 ± 0.16	1.61 ± 0.29	2.07 ± 0.00	1.59 ± 0.46
Gly	1.88 ± 0.21	2.07 ± 0.03	1.74 ± 0.05	2.48 ± 0.00	2.67 ± 0.13	2.69 ± 0.38
Ile	0.84 ± 0.12	0.80 ± 0.11	0.23 ± 0.02	2.60 ± 0.90	0.84 ± 0.00	1.18 ± 0.45
Leu	0.97 ± 0.29	1.18 ± 0.08	0.88 ± 0.06	0.63 ± 0.06	1.19 ± 0.00	1.29 ± 0.33
Met	1.23 ± 0.19	1.56 ± 0.18	1.47 ± 0.06	1.30 ± 0.16	1.36 ± 0.00	1.26 ± 0.10
Phe	2.98 ± 0.00	4.74 ± 0.90	0.89 ± 0.12	0.00 ± 0.00	0.84 ± 0.18	5.46 ± 0.00
Pro	6.41 ± 0.08	8.67 ± 1.06	5.02 ± 0.55	3.76 ± 0.00	0.00 ± 0.00	0.00 ± 0.00
Ser	0.38 ± 0.07	0.62 ± 0.05	0.82 ± 0.15	0.83 ± 0.05	0.59 ± 0.00	0.47 ± 0.11
Thr	2.00 ± 0.10	2.17 ± 0.08	1.97 ± 0.24	2.84 ± 0.22	2.03 ± 0.00	2.44 ± 0.24
Tyr	3.01 ± 0.09	1.77 ± 0.15	2.03 ± 0.19	0.00 ± 0.00	1.66 ± 0.01	1.94 ± 0.35
Val	0.72 ± 0.11	0.81 ± 0.29	0.53 ± 0.09	0.09 ± 0.04	0.16 ± 0.00	1.01 ± 0.00
GABA	1.14 ± 0.22	2.27 ± 0.30	3.23 ± 0.39	1.47 ± 0.12	1.30 ± 0.00	1.65 ± 0.07

Values represent mean ± SE (n = 3).

Supplementary Table S3. ^{13}C enrichment of organic acids and amino acids in wheat roots grown under nitrate (RN-[^{13}C]Pyr) or ammonium (RA-[^{13}C]Pyr) nutrition incubated with 10 mM [^{13}C]Pyr for 0.5, 2 and 6 h.

	[^{13}C]organic acid and [^{13}C]amino acid enrichment (%)					
	RN-^{13}C-Pyr			RA-^{13}C-Pyr		
	30 min	2 h	6 h	30 min	2 h	6 h
Pyr	81.22 ± 5.10	89.23 ± 1.29	80.92 ± 5.21	78.59 ± 5.29	75.15 ± 3.30	76.42 ± 1.95
Citrate	3.22 ± 1.22	10.87 ± 1.38	26.92 ± 1.32	11.61 ± 1.65	22.88 ± 0.52	47.22 ± 0.48
Isocitrate	2.08 ± 0.71	9.48 ± 0.00	11.59 ± 1.34	10.26 ± 0.56	17.90 ± 1.13	44.80 ± 0.48
2-OG	3.47 ± 0.04	16.28 ± 0.74	28.34 ± 1.42	8.59 ± 0.53	20.42 ± 0.30	32.27 ± 0.57
Succinate	0.38 ± 0.06	1.61 ± 0.21	3.82 ± 0.27	1.62 ± 0.15	2.99 ± 0.11	8.46 ± 0.40
Fumarate	1.61 ± 0.22	12.23 ± 0.72	24.72 ± 1.46	4.63 ± 0.44	13.26 ± 0.41	23.93 ± 0.42
Malate	0.47 ± 0.27	3.79 ± 0.42	12.86 ± 1.60	2.88 ± 0.52	11.25 ± 1.21	22.26 ± 0.56
Ala	10.72 ± 2.33	26.17 ± 1.10	27.21 ± 1.74	7.19 ± 0.80	8.33 ± 0.37	12.03 ± 0.27
Asn	0.38 ± 0.25	1.04 ± 0.26	5.45 ± 1.78	0.17 ± 0.00	1.28 ± 0.03	1.43 ± 0.22
Asp	1.98 ± 0.06	11.21 ± 1.01	21.60 ± 1.76	4.18 ± 0.68	7.99 ± 0.46	20.36 ± 0.22
Cys	0.04 ± 0.00	0.35 ± 0.00	0.12 ± 0.01	0.14 ± 0.09	0.44 ± 0.21	0.19 ± 0.11
Gln	0.67 ± 0.25	13.54 ± 0.77	21.70 ± 2.56	1.69 ± 0.52	8.03 ± 0.94	10.90 ± 2.19
Glu	3.52 ± 0.52	19.22 ± 0.83	30.69 ± 2.28	7.54 ± 0.50	19.60 ± 0.32	35.10 ± 0.27
Gly	0.00 ± 0.00	1.01 ± 0.00	0.44 ± 0.22	1.00 ± 0.00	1.77 ± 0.25	2.32 ± 0.33
His	0.00 ± 0.00	0.00 ± 0.00	0.00 ± 0.00	4.66 ± 1.59	2.58 ± 0.89	2.39 ± 0.93
Ile	0.33 ± 0.18	0.57 ± 0.26	0.19 ± 0.06	0.21 ± 0.11	0.22 ± 0.03	0.65 ± 0.10
Leu	1.44 ± 0.93	0.29 ± 0.00	0.18 ± 0.10	0.34 ± 0.07	0.18 ± 0.05	1.00 ± 0.20
Lys	12.95 ± 6.70	0.00 ± 0.00	0.00 ± 0.00	4.35 ± 0.00	0.00 ± 0.00	3.16 ± 1.47
Met	0.00 ± 0.00	0.00 ± 0.00	0.00 ± 0.00	1.07 ± 0.00	0.82 ± 0.47	1.08 ± 0.16
Phe	2.59 ± 0.00	2.21 ± 0.93	7.64 ± 0.13	3.00 ± 0.72	4.61 ± 0.93	5.58 ± 0.27
Ser	0.17 ± 0.00	0.99 ± 0.28	2.77 ± 0.16	0.61 ± 0.48	0.35 ± 0.07	4.33 ± 0.07
Tyr	0.00 ± 0.00	0.00 ± 0.00	3.36 ± 1.49	3.41 ± 2.53	2.83 ± 1.35	1.86 ± 0.39
Val	0.55 ± 0.29	1.42 ± 0.20	0.86 ± 0.33	0.38 ± 0.28	0.42 ± 0.05	1.16 ± 0.21
GABA	0.78 ± 0.52	3.89 ± 0.92	15.50 ± 0.55	0.68 ± 0.08	4.34 ± 0.24	14.66 ± 0.58

Values represent mean ± SE (n = 3).

Supplementary Table S4. ^{13}C enrichment distribution among each mass peak (from m+1 to m+4) of organic acids and amino acids after [^{13}C]Pyr-incubation of roots of wheat plants grown under nitrate (RN-[^{13}C]Pyr) or ammonium (RA-[^{13}C]Pyr) nutrition.

			[^{13}C]organic acid and [^{13}C]amino acid enrichment (%)					
			RN-[^{13}C]Pyr			RA-[^{13}C]Pyr		
	C atoms per molecule	Mass peak	30 min	2 h	6 h	30 min	2 h	6 h
Pyr	C3	m+1	0.13 ± 0.03	0.24 ± 0.00	1.03 ± 0.21	0.23 ± 0.04	0.57 ± 0.04	1.16 ± 0.12
		m+2	1.19 ± 0.06	1.32 ± 0.03	1.65 ± 0.03	1.19 ± 0.07	1.38 ± 0.02	1.79 ± 0.05
		m+3	79.90 ± 5.07	87.68 ± 1.26	78.24 ± 5.45	77.17 ± 5.25	73.19 ± 3.33	73.47 ± 2.12
Citrate	C6	m+1	0.82 ± 0.46	1.54 ± 0.20	4.58 ± 0.61	1.92 ± 0.91	3.80 ± 0.20	7.76 ± 0.34
		m+2	1.16 ± 0.32	4.67 ± 0.55	10.85 ± 0.56	4.88 ± 0.44	9.28 ± 0.19	18.48 ± 0.23
		m+3	0.80 ± 0.29	2.92 ± 0.39	7.20 ± 0.42	3.10 ± 0.23	6.12 ± 0.11	13.00 ± 0.09
		m+4	0.44 ± 0.16	1.75 ± 0.23	4.29 ± 0.30	1.72 ± 0.12	3.68 ± 0.05	7.98 ± 0.05
Isocitrate	C6	m+1	1.11 ± 0.41	2.71 ± 0.00	2.49 ± 1.16	1.94 ± 0.36	2.79 ± 0.77	8.04 ± 0.28
		m+2	0.84 ± 0.25	3.43 ± 0.00	4.69 ± 0.28	3.70 ± 0.52	6.97 ± 0.09	17.20 ± 0.18
		m+3	0.00 ± 0.00	1.94 ± 0.00	3.15 ± 0.25	2.95 ± 0.50	5.26 ± 0.20	12.18 ± 0.07
		m+4	0.12 ± 0.05	1.40 ± 0.00	1.25 ± 0.21	1.68 ± 0.08	2.88 ± 0.08	7.38 ± 0.06
2-OG	C5	m+1	0.24 ± 0.06	2.75 ± 0.21	6.95 ± 0.02	1.01 ± 0.09	3.29 ± 0.15	6.07 ± 0.12
		m+2	1.96 ± 0.12	9.52 ± 0.39	13.45 ± 0.81	5.23 ± 0.29	10.94 ± 0.13	15.85 ± 0.32
		m+3	0.87 ± 0.05	2.62 ± 0.02	5.30 ± 0.35	1.64 ± 0.14	4.10 ± 0.08	6.55 ± 0.11
		m+4	0.40 ± 0.03	1.40 ± 0.13	2.64 ± 0.25	0.72 ± 0.04	2.09 ± 0.04	3.79 ± 0.09
Succinate	C4	m+1	0.09 ± 0.08	2.32 ± 0.23	7.64 ± 0.21	0.27 ± 0.12	2.27 ± 0.07	5.47 ± 0.15
		m+2	1.10 ± 0.09	7.13 ± 0.29	11.34 ± 0.78	3.14 ± 0.20	7.70 ± 0.21	12.29 ± 0.20
		m+3	0.31 ± 0.05	1.99 ± 0.13	4.24 ± 0.35	0.83 ± 0.11	2.28 ± 0.10	4.43 ± 0.08
		m+4	0.14 0.03	0.80 0.06	1.50 0.13	0.39 0.06	1.01 0.04	1.74 0.04
Fumarate	C4	m+1	0.12 ± 0.06	0.60 ± 0.06	1.32 ± 0.06	0.68 ± 0.03	1.01 ± 0.10	2.80 ± 0.14
		m+2	0.24 ± 0.01	0.73 ± 0.09	1.76 ± 0.12	0.59 ± 0.10	1.15 ± 0.00	3.55 ± 0.15
		m+3	0.03 ± 0.01	0.22 ± 0.06	0.55 ± 0.07	0.27 ± 0.02	0.60 ± 0.01	1.53 ± 0.08
		m+4	0.00 ± 0.00	0.06 ± 0.00	0.20 ± 0.04	0.08 ± 0.02	0.22 ± 0.01	0.59 ± 0.04
Malate	C4	m+1	0.22 ± 0.11	1.04 ± 0.08	3.75 ± 0.38	0.84 ± 0.36	3.84 ± 0.41	7.13 ± 0.25
		m+2	0.19 ± 0.10	1.65 ± 0.15	5.28 ± 0.63	0.94 ± 0.15	4.21 ± 0.45	9.01 ± 0.24
		m+3	0.11 ± 0.00	0.78 ± 0.14	2.59 ± 0.41	0.71 ± 0.07	2.21 ± 0.25	4.23 ± 0.10
		m+4	0.00 ± 0.00	0.31 ± 0.05	1.24 ± 0.20	0.39 ± 0.03	0.99 ± 0.11	1.88 ± 0.03
Ala	C3	m+1	0.11 ± 0.02	1.30 ± 0.14	3.96 ± 0.18	0.45 ± 0.33	0.73 ± 0.11	1.80 ± 0.06
		m+2	0.31 ± 0.04	1.69 ± 0.16	3.17 ± 0.25	0.37 ± 0.11	0.74 ± 0.06	1.77 ± 0.08
		m+3	10.30 ± 2.36	23.18 ± 0.80	20.08 ± 1.48	6.52 ± 0.72	6.87 ± 0.36	8.46 ± 0.14
Glu	C5	m+1	0.63 ± 0.11	2.44 ± 0.17	6.50 ± 0.15	0.85 ± 0.22	2.92 ± 0.12	5.87 ± 0.10
		m+2	1.83 ± 0.22	10.32 ± 0.37	13.78 ± 1.15	4.05 ± 0.19	9.68 ± 0.11	16.13 ± 0.18
		m+3	0.72 ± 0.13	4.32 ± 0.18	6.88 ± 0.62	1.77 ± 0.11	4.61 ± 0.07	8.40 ± 0.07
		m+4	0.35 0.07	2.13 0.11	3.53 0.37	0.86 0.07	2.39 0.05	4.69 0.01
Gln	C5	m+1	0.00 ± 0.00	2.21 ± 0.37	4.49 ± 0.39	0.00 ± 0.00	1.11 ± 0.19	1.28 ± 0.68
		m+2	0.63 ± 0.21	6.75 ± 0.11	9.95 ± 0.98	0.83 ± 0.23	3.95 ± 0.32	5.44 ± 0.84
		m+3	0.09 ± 0.00	3.18 ± 0.17	4.89 ± 0.81	0.48 ± 0.23	2.09 ± 0.27	2.78 ± 0.50
		m+4	0.03 ± 0.00	1.39 ± 0.12	2.37 ± 0.43	0.38 ± 0.07	0.88 ± 0.19	1.40 ± 0.30
Asp	C4	m+1	0.79 ± 0.07	3.41 ± 0.31	6.65 ± 0.26	2.00 ± 0.26	3.57 ± 0.08	6.34 ± 0.22
		m+2	0.73 ± 0.04	4.73 ± 0.35	8.42 ± 0.79	1.23 ± 0.18	3.81 ± 0.08	7.84 ± 0.20
		m+3	0.33 ± 0.03	2.12 ± 0.23	4.56 ± 0.48	0.67 ± 0.25	1.32 ± 0.00	4.28 ± 0.11
		m+4	0.13 ± 0.01	0.95 ± 0.12	1.98 ± 0.24	0.28 ± 0.11	0.53 ± 0.00	1.91 ± 0.08
Asn	C4	m+1	0.20 ± 0.09	0.41 ± 0.07	1.59 ± 0.52	0.00 ± 0.00	0.44 ± 0.04	0.39 ± 0.19
		m+2	0.22 ± 0.00	0.37 ± 0.09	2.41 ± 0.73	0.00 ± 0.00	0.43 ± 0.02	0.66 ± 0.06
		m+3	0.12 ± 0.00	0.18 ± 0.06	0.97 ± 0.37	0.09 ± 0.00	0.26 ± 0.03	0.30 ± 0.01
		m+4	0.02 ± 0.00	0.08 ± 0.03	0.48 ± 0.17	0.09 ± 0.00	0.14 ± 0.03	0.08 ± 0.03
GABA	C4	m+1	0.10 ± 0.00	0.82 ± 0.40	5.24 ± 0.02	0.10 ± 0.04	0.56 ± 0.09	3.09 ± 0.07
		m+2	0.55 ± 0.39	2.27 ± 0.33	7.05 ± 0.29	0.42 ± 0.04	2.65 ± 0.12	8.02 ± 0.37
		m+3	0.18 ± 0.00	0.63 ± 0.13	2.37 ± 0.12	0.10 ± 0.02	0.79 ± 0.04	2.58 ± 0.11
		m+4	0.08 ± 0.00	0.17 ± 0.06	0.84 ± 0.13	0.05 ± 0.01	0.33 ± 0.02	0.96 ± 0.04

For each molecule, the gray scale ilustrates the highest (dark grey) and the lowest (white) enrichment value under each N nutrition. Values represent mean ± SE (n = 3).

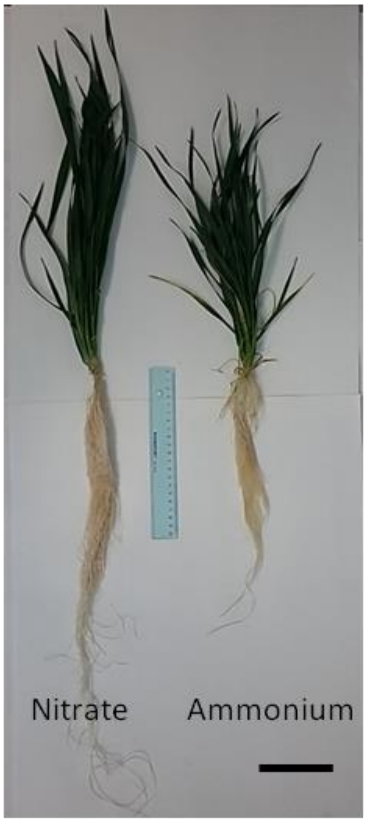

Supplementary Figure S1. Six-week old wheat plants grown under nitrate or ammonium nutrition. Scale bar indicates 10 cm length.

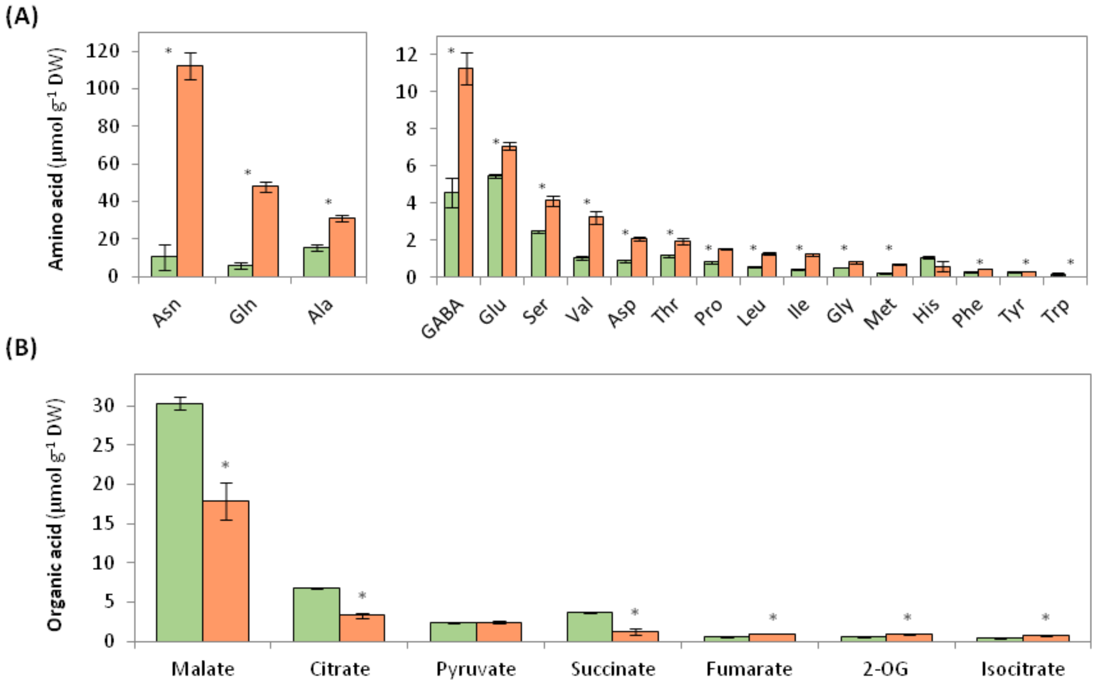

Supplementary Figure S2. Total (A) amino acids and (B) organic acids from wheat roots grown under nitrate (RN; green) or ammonium (RA; orange) nutrition. Values represent mean ± SE (n = 3). Asterisk (*) indicates significant differences between N nutritions ($p < 0.05$).

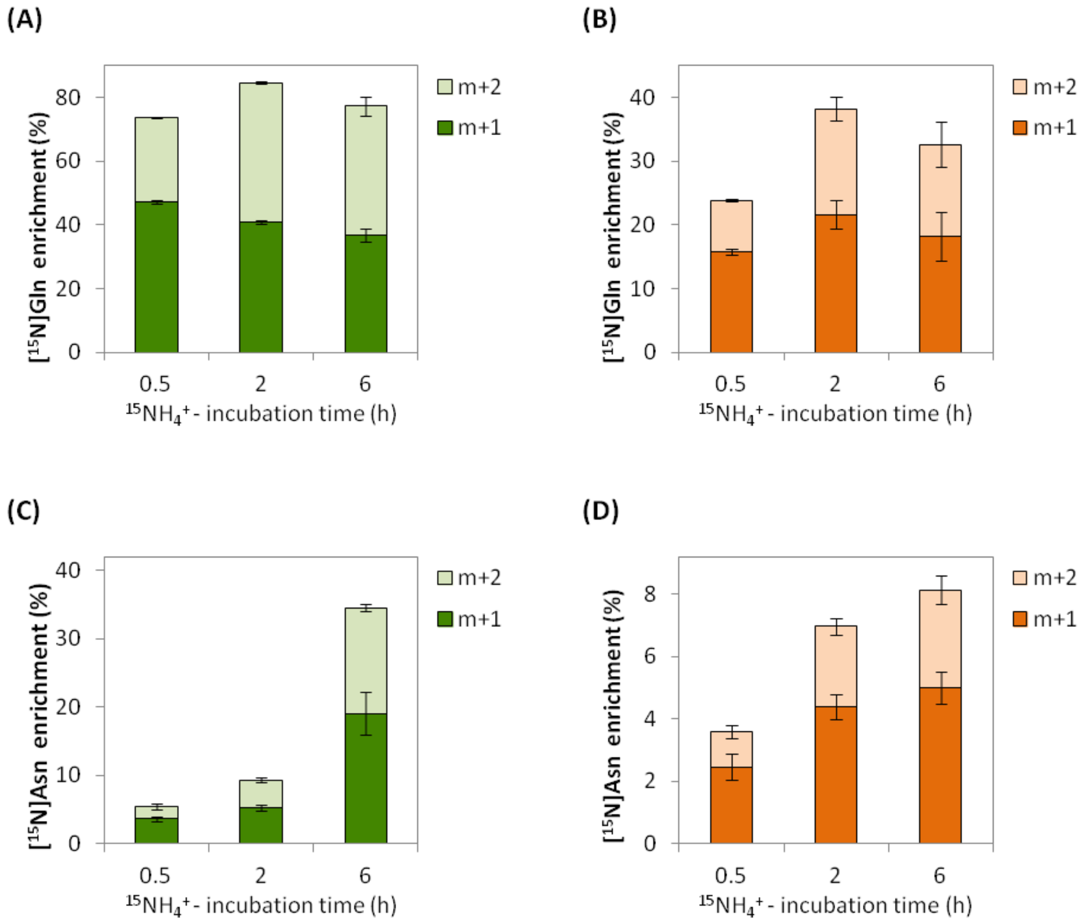

Supplementary Figure S3. Label distribution in one or two N atoms of amides after $^{15}NH_4$-incubation of wheat roots. ^{15}N enrichment of one N atom (given by m+1 value) or two N atoms (given by m+2 value) for [^{15}N]Gln and [^{15}N]Asn in wheat plants grown under (A, C) nitrate or (B, D) ammonium nutrition. Values represent mean ± SE (n = 3).

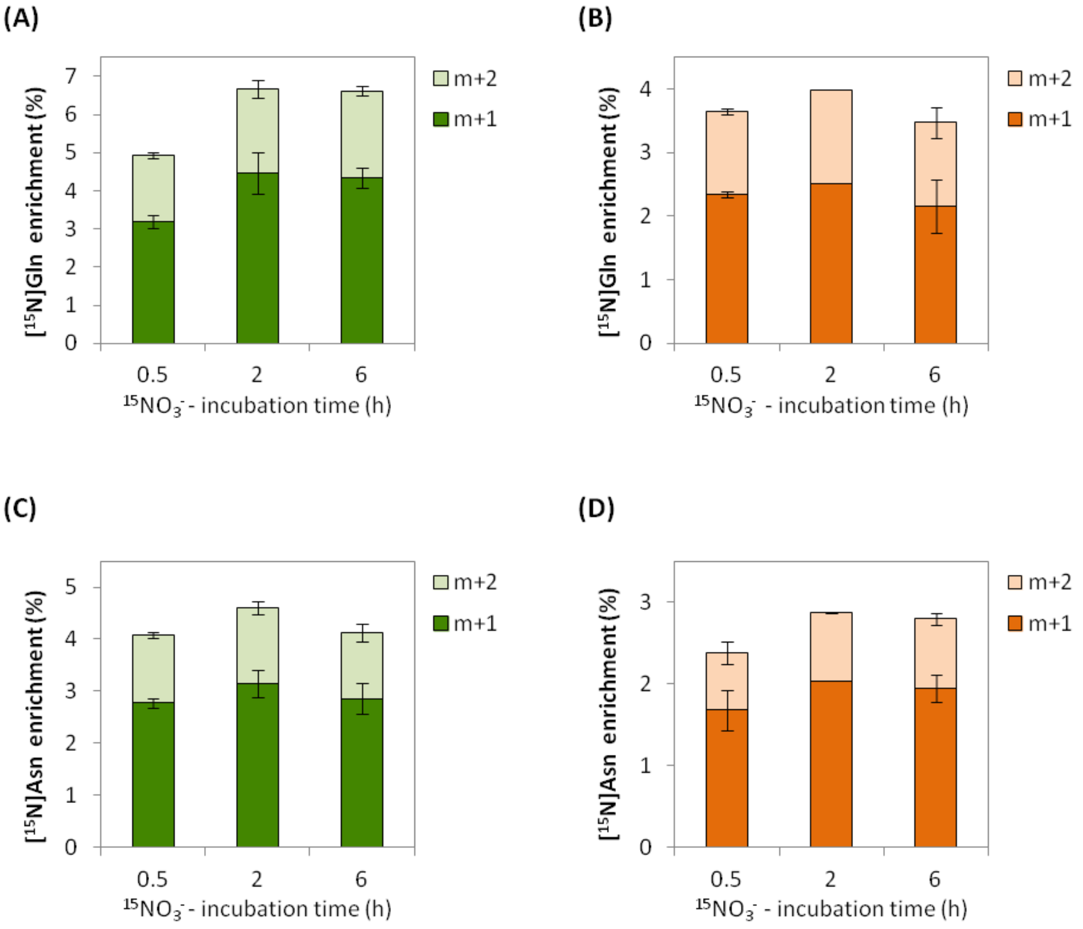

Supplementary Figure S4. Label distribution in one or two N atoms of amides after $^{15}NO_3$-incubation of wheat roots. ^{15}N enrichment of one N atom (given by m+1 value) or two N atoms (given by m+2 value) for [^{15}N]Gln and [^{15}N]Asn in wheat plants grown under (A, C) nitrate or (B, D) ammonium nutrition. Values represent mean ± SE (n = 3).

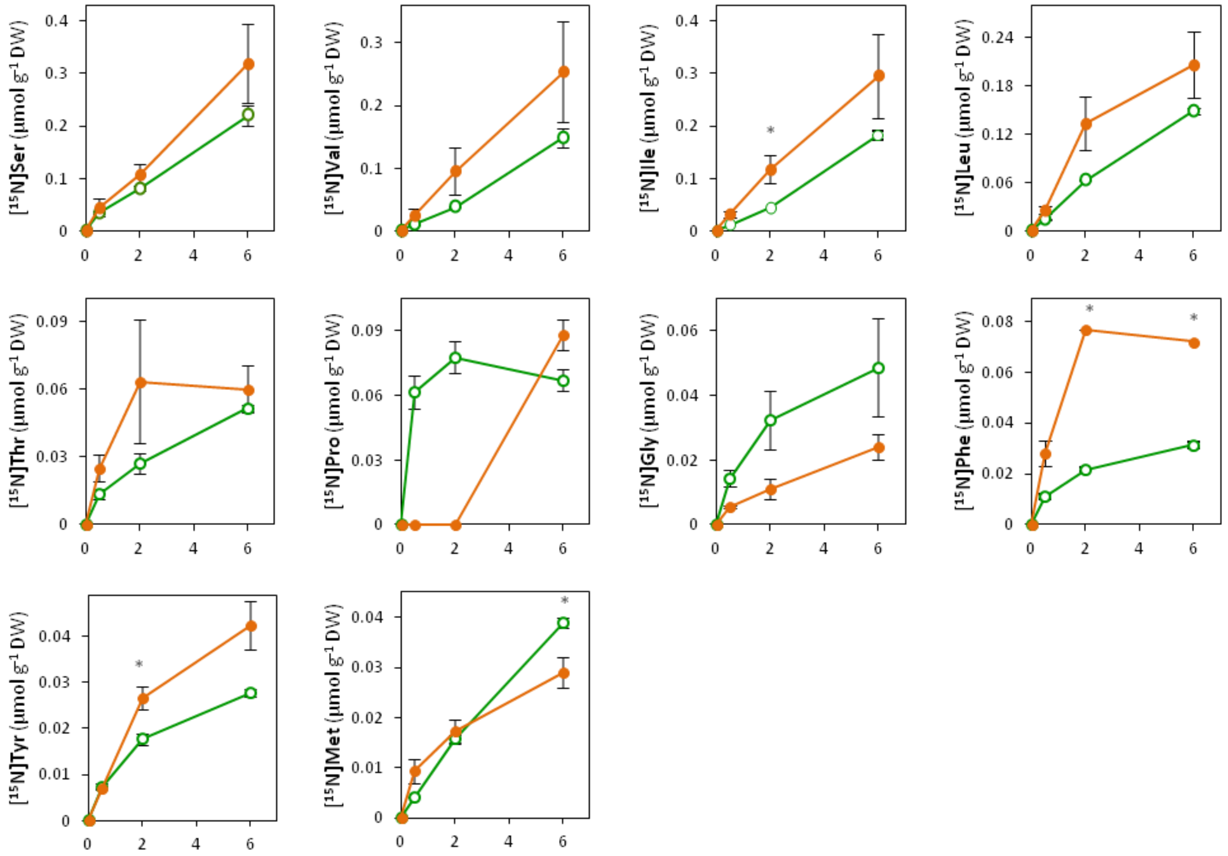

Supplementary Figure S5. Minor [^{15}N]amino acid contents (µmol g^{-1} DW) after $^{15}NH_4^+$-incubation of roots of wheat plants previously grown under nitrate (green open circle) or ammonium (orange closed circle) nutrition. X-axis indicates the incubation times: 0.5, 2, and 6 h. Values represent mean ± SE (n = 3). Asterisk (*) indicates significant differences between N nutritions ($p < 0.05$).

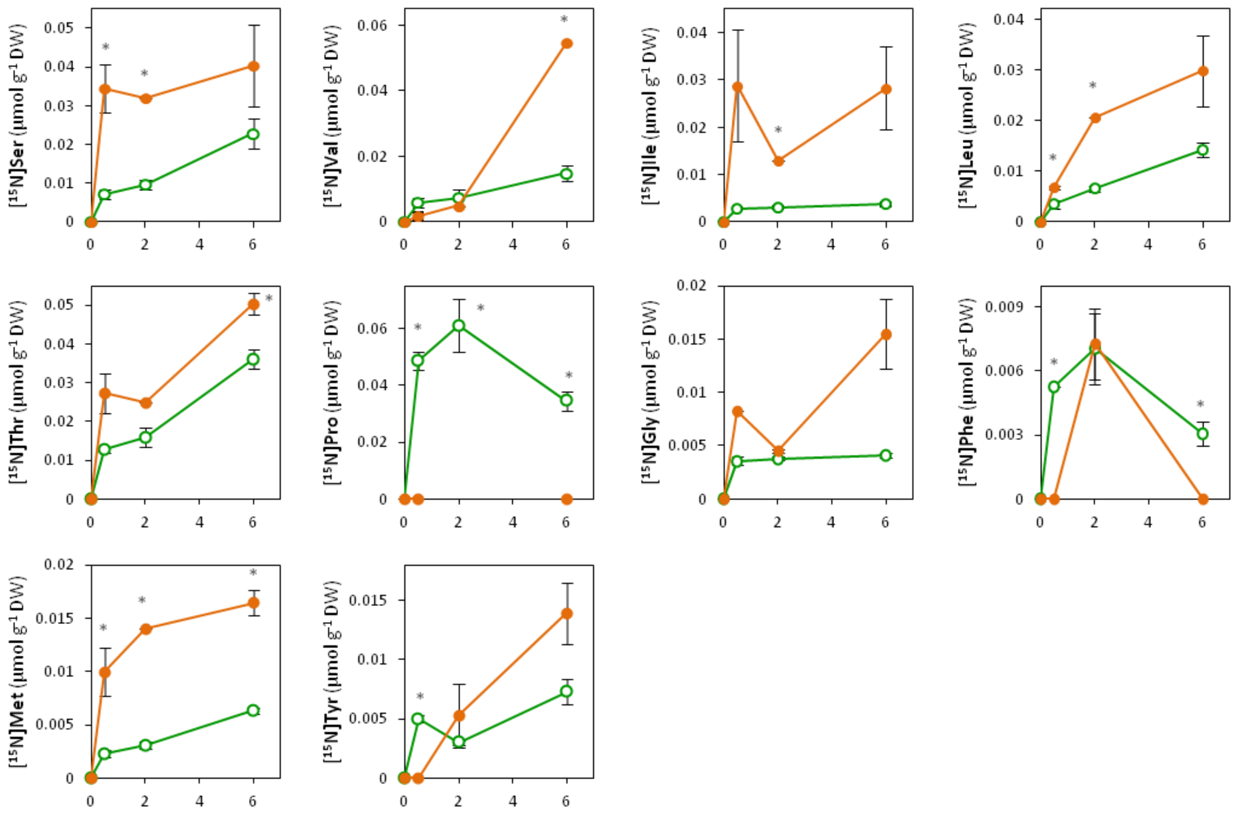

Supplementary Figure S6. Minor $[^{15}N]$amino acid contents (µmol g^{-1} DW) after $^{15}NO_3^-$ -incubation of roots of wheat plants previously grown under nitrate (green open circle) or ammonium (orange closed circle) nutrition. X-axis indicates the incubation times: 0.5, 2, and 6 h. Values represent mean ± SE (n = 3). Asterisk (*) indicates significant differences between N nutritions ($p < 0.05$).

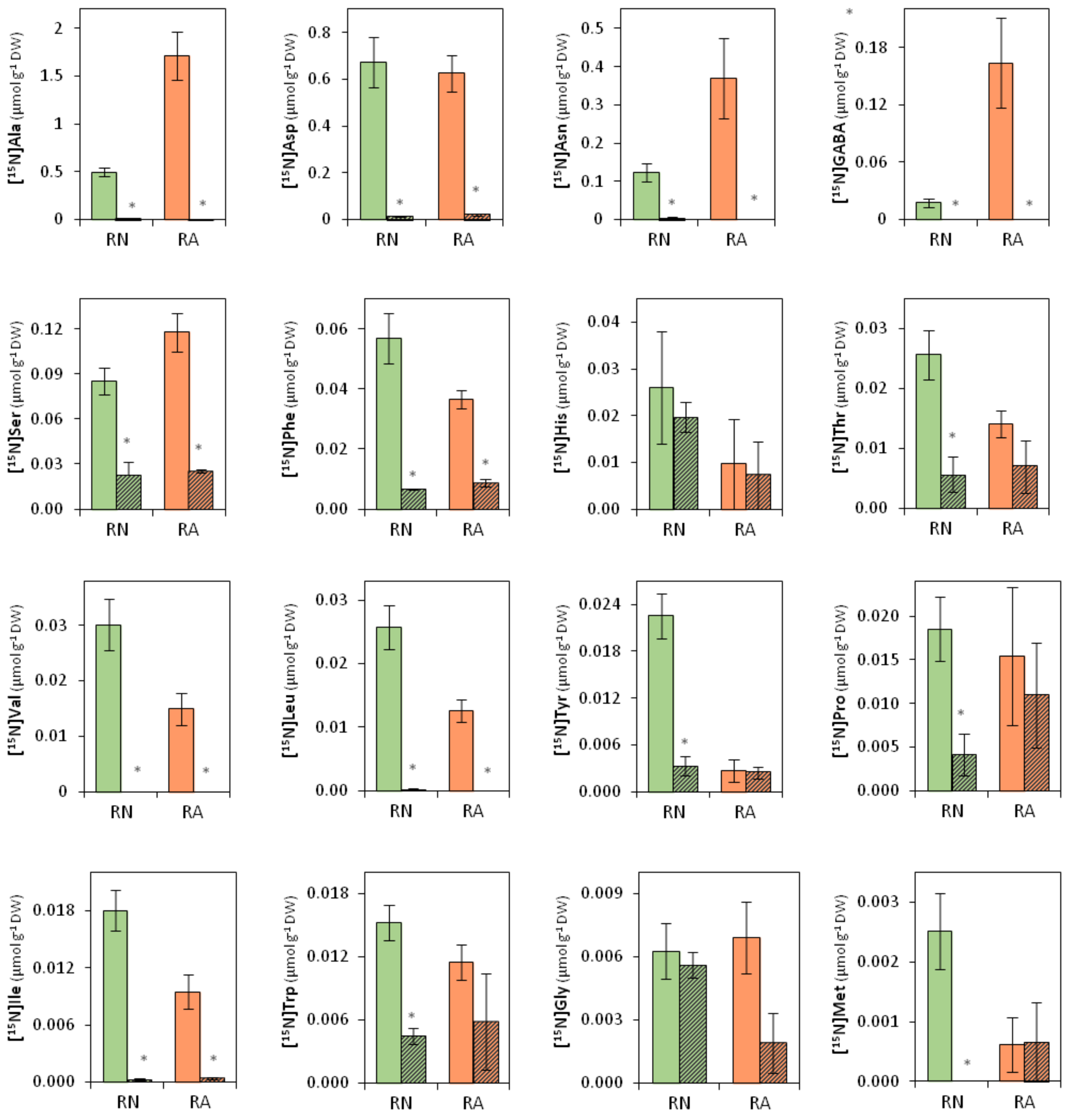

Supplementary Figure S7. Contents of other labelled amino acids in wheat roots grown under nitrate (RN; green) or ammonium (RA; orange) nutrition incubated with 10 mM $^{15}NH_4^+$ for 30 minutes in absence (plain bar) or in presence of inhibitors MSX and AZA (striped bar). Values represent mean ± SE (n = 3). Asterisk (*) indicates significant differences between N nutritions ($p < 0.05$).

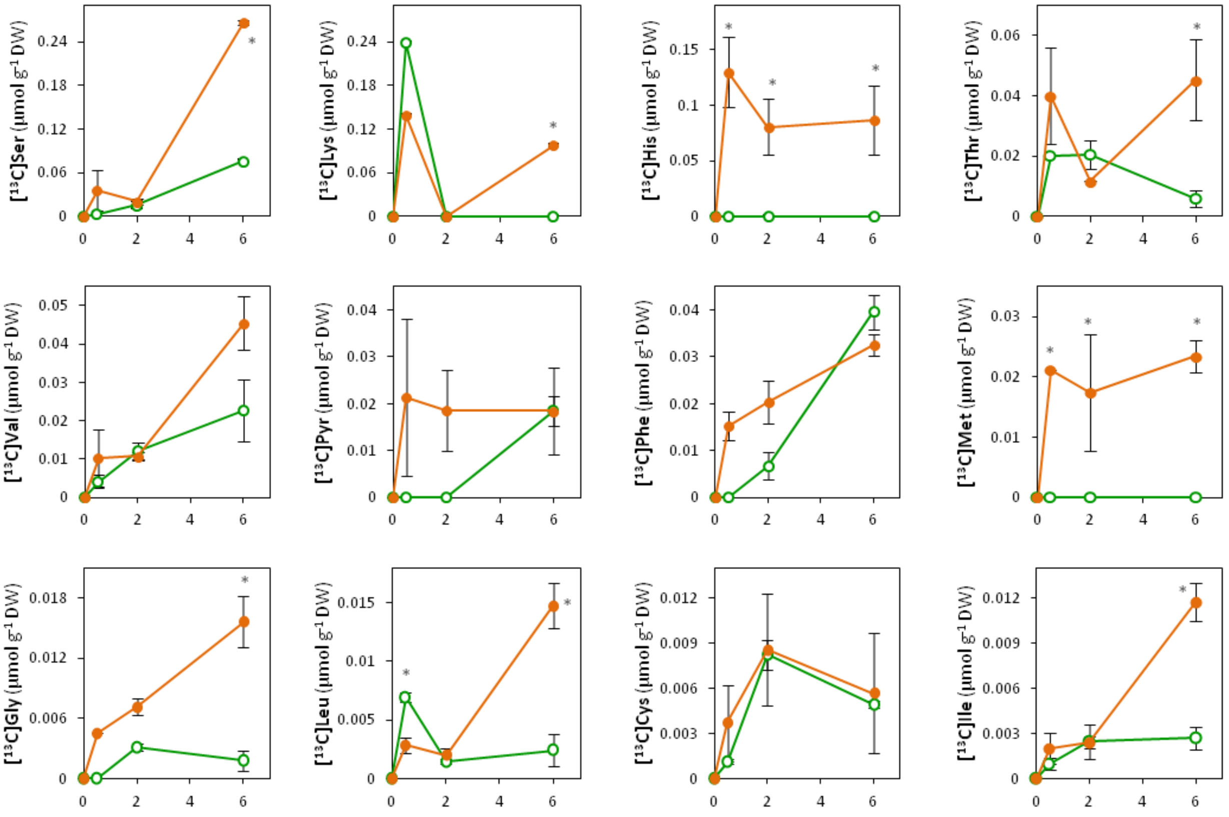

Supplementary Figure S8. Minor [^{15}N]amino acid and [^{13}C]organic acid contents (µmol g^{-1} DW) after [^{13}C]Pyr -incubation of roots of plants previously grown under nitrate (green open circle) or ammonium (orange closed circle) nutrition. X-axis indicates the incubation times: 0.5, 2 and 6 h. Values represent mean ± SE (n = 3). Asterisk (*) indicates significant differences between N nutritions ($p < 0.05$).
